# JAK-centric explainable few-shot gene-expression diagnosis framework for alopecia via MultiPLIER priors and relation-style set-to-set comparison

**DOI:** 10.3389/fmolb.2025.1753206

**Published:** 2026-01-12

**Authors:** Nanlan Yu, Ling Ran, Xinrong Gong, Junfei Teng, Shulei Liu, Zhiqiang Song

**Affiliations:** 1 Department of Dermatology, The First Affiliated Hospital (Southwest Hospital), Army Medical University, Chongqing, China; 2 Chongqing Key Laboratory for the Mechanism and Intervention of Cancer Metastasis, Chongqing University Cancer Hospital, Chongqing University, Chongqing, China; 3 School of Engineering, Huaqiao University, Quanzhou, China; 4 Department of Dermatology of Jiangbei Campus, The First Affiliated Hospital of Army Medical University, Chongqing, China

**Keywords:** alopecia areata, few-shot learning, gene expression, MultiPLIER, permutation-invariant aggregation, relation network, transfer learning

## Abstract

Alopecia areata (AA) and androgenetic alopecia (AGA) both present with hair loss but require different therapies, and reliable biomarkers to guide treatment remain lacking. We integrated bulk and single-cell RNA-seq to compare JAK–STAT signaling in AA versus AGA. In AA, 257 immune-enriched differentially expressed genes (DEGs) were identified; WGCNA and consensus machine learning (LASSO, SVM-RFE, random forest) yielded six candidate hub genes, and external validation narrowed these to four key genes–granzyme A (GZMA), interleukin-2 receptor β (IL2RB) and γ (IL2RG) chains, and eomesodermin (EOMES). Building on these biology-anchored features, we introduced an interpretable few-shot deep learning classifier as an explainable AI alternative to a nomogram: bulk expression profiles are projected onto pathway/cell-type–aligned MultiPLIER latent variables (a frozen prior), the latent channels are re-weighted via element-wise multiplication with the expression levels of the key hub genes, and a Relation-style set-to-set comparator then aggregates support–query similarities (Hadamard mapping + permutation-invariant aggregation) before a shallow head predicts AA versus control. This prior-informed approach enables robust discrimination under limited sample conditions while retaining mechanistic interpretability, thereby exemplifying a next-generation XAI solution for small-cohort genomic diagnosis. Cross-database functional annotation and wet-lab validation (RT-qPCR and Western blot) in independent AA/AGA/healthy scalp samples confirmed that the IL2RB/IL2RG–EOMES–GZMA axis is selectively activated at both mRNA and protein levels in AA. Single-cell analysis localized GZMA to cytotoxic T cells and IL2RG to proliferating lymphocytes, outlining an ${\text{EOMES}}^{+}$ ${\text{CD}8}^{+}$ T-cell GZMA–IL2RB/IL2RG cytotoxic loop driving JAK–STAT hyperactivation in AA. Drug–gene network analysis linked these targets to JAK inhibitors and cyclosporine. AGA showed no comparable JAK–STAT perturbation, consistent with its androgen-centric biology. In summary, this four-gene loop provides a non-invasive AA biomarker and a tractable target for precision JAK blockade, while the proposed few-shot framework offers a general, prior-driven alternative to nomograms for transcriptomic diagnosis in small cohorts, illustrating an XAI-driven diagnostic approach for precision medicine.

## 1 Introduction

AA and AGA are the two most common hair loss disorders and have markedly different clinical features and epidemiological impacts (Zhou et al., 2021). AA presents as sudden-onset, non-scarring hair loss caused by breakdown of the hair follicle’s immune privilege and a cytotoxic T-cell-mediated autoimmune attack (Dai et al., 2021); its lifetime prevalence is 0.1%–2%, with considerable psychosocial burden (Strazzulla et al., 2018). By contrast, AGA is driven by androgen-dependent miniaturization of scalp hair follicles (especially in frontal and vertex regions) via androgen receptor signaling in dermal papilla cells, with only limited inflammatory involvement (Anzai et al., 2019). AA can progress to alopecia totalis or universalis, whereas AGA usually arises in adulthood and is epidemiologically linked to cardiometabolic comorbidities (Gonul et al., 2018). These distinct clinical courses and systemic associations underscore the need for disease-specific biomarkers and targeted therapies.

The pathophysiology of AA is driven by dysregulated activation of the JAK/STAT (Janus kinase/Signal Transducer and Activator of Transcription) signaling pathway (Dai et al., 2021; Haughton et al., 2023). A key downstream element is the IFN- γ /IL-15 (interferon- γ /interleukin-15) axis, which persistently recruits and sustains ${\text{CD}8}^{+}{\text{NKG}2\text{D}}^{+}$ cytotoxic T cells around anagen hair follicles, breaking immune privilege and inducing keratinocyte apoptosis, ultimately causing hair loss (Dai et al., 2021; Haughton et al., 2023). JAK inhibitors (e.g., baricitinib, tofacitinib) have shown significant hair regrowth in AA clinical trials by blocking STAT1/STAT3 phosphorylation and inhibiting effector T cell function (Wada-Irimada et al., 2025). In AGA, by contrast, pathogenesis is primarily driven by the dihydrotestosterone (DHT)–androgen receptor (AR) axis, which increases NADPH oxidase (NOX)-mediated oxidative stress and pro-apoptotic molecules (e.g., BAX), leading to follicular miniaturization and premature catagen entry, with far less inflammatory infiltration than AA. Although studies have noted elevated STAT3 in AGA scalps, it shows no strong correlation with clinical severity, and immune-targeted therapies have limited efficacy (Hassan et al., 2022). Thus, first-line AGA treatments remain anti-androgenic drugs (finasteride, minoxidil), underscoring fundamental differences in molecular pathophysiology and treatment strategy between AA and AGA (Ocampo-Garza et al., 2019).

To date, AA research has focused mainly on STAT-mediated cytokine signaling and related interventions, whereas AGA studies have largely been limited to androgen pathways or a few isolated molecular markers (Haughton et al., 2023; Wada-Irimada et al., 2025; Sardana et al., 2018). Significant knowledge gaps remain. First, there have been no comprehensive, side-by-side comparisons of the JAK-STAT signaling networks in AA versus AGA, leaving the immunological similarities and differences between the two diseases incompletely explained—AA’s pathology features excessive T cell-driven JAK-STAT activation, whereas AGA studies have historically under emphasized inflammatory pathways. Second, cross-disease multi-omics studies are scarce, impeding the identification of common versus disease-specific hub genes and hindering the development of precise diagnostics and targeted therapies (Ocampo-Garza et al., 2019; Sardana et al., 2018). Finally, in clinical translation, reliance on linear models (e.g., logistic regression nomograms) persists, yet such models perform poorly in small-cohort, high-dimensional transcriptomic settings–precisely where robust, mechanism-anchored representations and sample-efficient learning are most needed (Clough and Barrett, 2016; Ritchie et al., 2015). These limitations underscore the need for advanced machine learning frameworks that can operate on limited data while maintaining transparency. In other words, an explainable AI approach is required to meet these challenges in a modern Healthcare 4.0 context.

Clarifying these mechanistic differences is critical for developing precise interventions: for AA, immune-targeted therapies (e.g., JAK inhibition) should be prioritized, whereas for AGA, treatments should focus on androgen-dependent signaling. Clearly distinguishing the molecular features of the two disorders will not only accelerate the discovery of non-invasive biomarkers but also lay the groundwork for innovative, mechanism-based treatments. Accordingly, in this study we performed a multi-omics integrative analysis to systematically compare the JAK-STAT networks in AA and AGA, identify disease-specific hub genes, and evaluate their potential utility in early diagnosis and personalized treatment.

To meet these challenges, we integrated bulk and single-cell RNA-seq data to map JAK-STAT biology in AA and AGA, while retaining a biologically grounded feature-selection pipeline (DEGs → WGCNA → PPI/network prioritization → consensus machine learning). Building on this foundation, we replaced the traditional nomogram with an interpretable few-shot deep learning framework, embodying an explainable AI approach, that (i) projects each bulk expression profile onto pathway/cell-type–aligned latent variables using a frozen MultiPLIER prior (Guo et al., 2023), (ii) amplifies AA-relevant signals via weighting latent channels by the hub gene set, and (iii) conducts a Relation Network–style set-to-set comparison between support and query samples (Hadamard similarity mapping with permutation-invariant aggregation) followed by a shallow classifier. This prior-driven, small-sample–oriented design preserves mechanistic interpretability while improving classification performance under limited sample conditions, effectively addressing the performance–interpretability trade-off emphasized in XAI 2.0 for Healthcare 4.0. Finally, we situate the AA-specific hub genes within an immune-cytotoxic JAK-STAT loop and contrast this mechanism against AGA’s androgen-centric, low-inflammation landscape, highlighting distinct pathogenic circuits in each disease.

Specifically, the primary contributions of this work are:

1. Prior-driven, interpretable representations for small cohorts. Bulk gene expression profiles are projected onto pathway- and cell type–aligned latent variables using a frozen MultiPLIER prior, and then these latent dimensions are re-weighted according to the expression of the consensus hub genes. This approach produces a mechanism-anchored embedding that reduces data requirements while preserving biological interpretability and context relevance.
2. Relation-style set-to-set comparison as a nomogram alternative. Instead of using a linear nomogram, our method performs full support–query matching via a Hadamard similarity map with permutation-invariant aggregation, followed by a shallow classifier (i.e., a Relation Network–style few-shot comparator tailored to transcriptomic data). This design is both parameter-efficient and sample-efficient for small cohorts, and its case-based matching mechanism offers an intuitive interpretation of predictions.
3. End-to-end, biology-grounded pipeline with multi-omics and experimental validation. We maintain the robust feature-selection backbone and couple it to the few-shot classifier, then validate the pipeline across bulk and single-cell data and further confirm AA-specific activation of the IL2RB/IL2RG–EOMES–GZMA axis at both mRNA and protein levels in independent scalp samples. This approach localizes the ${\text{EOMES}}^{+}$ ${\text{CD}8}^{+}$ cytotoxic loop driving JAK–STAT hyperactivation in AA, in contrast to AGA’s androgen-centric, low-inflammation landscape. In doing so, it “closes the loop” from mechanistic insight to diagnosis and provides a prior-informed explainable alternative to linear models for limited-sample settings.

## 2 Methods

### 2.1 Data sources

Transcriptomic sequencing data for AA and AGA were obtained from the Gene Expression Omnibus (GEO) database (Langfelder and Horvath, 2008). Specifically, the training set data for AA came from the GSE68801 dataset (60 AA samples and 36 normal samples), while the validation set data came from the GSE45512 dataset (five AA samples and five normal samples). Additionally, transcriptome data for AGA were obtained from the GSE36169 dataset (five AGA samples and five normal samples), and the single-cell RNA sequencing (scRNA-seq) data of AA was from GSE212447, which included five AA samples for subsequent analysis.

### 2.2 Data preprocessing

For GSE68801 (AA, GPL570), GSE45512 (AA validation, GPL570), and GSE36169 (AGA, GPL96), we obtained either raw CEL files or processed series matrix files from GEO. When CEL files were available, microarrays were processed using the RMA pipeline (background correction, quantile normalization, and probe summarization). For series matrices, we verified that expression values were already $\log_{2}$ -transformed and quantile-normalized according to the original submitter’s methods. Probe IDs were mapped to HGNC gene symbols using the platform annotation, and if multiple probes mapped to the same gene, their values were collapsed by taking the median. To avoid label leakage, only lesional AA samples and healthy controls from GSE68801 were used for training (non-lesional AA samples were excluded).

### 2.3 Key gene selection workflow

#### 2.3.1 Differential expression analysis

Differential Expression Analysis: We used the limma package (v3.54.2) in R to identify differentially expressed genes (DEGs) between AA lesional and control samples in GSE68801 (Li et al., 2024). Genes with p<0.05 and |log⁡2 fold change|>0.5 were considered significantly differentially expressed. A volcano plot and heatmap were then generated to visualize the DEGs’ distribution patterns.

#### 2.3.2 Weighted gene co-expression network analysis (WGCNA)

Weighted Gene Co-expression Network Analysis (WGCNA): We applied WGCNA (v1.72.1) Szklarczyk et al. (2021); Shannon et al. (2003) on the GSE68801 expression matrix (all samples) to identify gene co-expression modules, using disease status (AA vs. control) as the trait. To ensure reliable results, we first performed sample clustering to check data correlations and remove any outlier samples. We then chose a soft-threshold power based on the scale-free topology criterion to optimize the network’s fit, and used it to construct a gene co-expression network with hierarchical clustering, yielding distinct gene modules. Next, Pearson correlation analysis was conducted between each module’s eigenexpression and the disease trait, and the modules with the strongest positive and negative correlations with AA were identified. The genes from these highly associated modules were selected for further investigation.

#### 2.3.3 Functional enrichment analysis

Functional Enrichment Analysis: We performed functional enrichment analysis on the intersecting gene set using the R package clusterProfiler (v4.6.2) (Bodinier et al., 2023; Narala et al., 2021). This included Gene Ontology (GO) enrichment—covering the three GO categories of cellular component (CC), biological process (BP), and molecular function (MF)—as well as Kyoto Encyclopedia of Genes and Genomes (KEGG) pathway analysis. The enrichment results for GO and KEGG were visualized with appropriate plots.

#### 2.3.4 Constructing PPI network based on the correlation analysis of the JAK family genes

First, correlation analysis was performed between specific gene expression profiles and JAK family genes, with Pearson correlation coefficients and p values used to assess linear relationships. Genes meeting the screening criteria ( | correlation coefficient |> 0.6 and p < 0.05) were retained. A protein-protein interaction (PPI) network was then constructed using the STRING database and visualized with Cytoscape (V 3.9.1) (Szklarczyk et al., 2021; Shannon et al., 2003). The top 20 hub genes were predicted using five topological analysis algorithms from the cytoHubba plugin, and the intersection analysis results were presented using an UpSet plot.

#### 2.3.5 Consensus machine-learning feature selection

To enhance the accuracy and reproducibility of high-dimensional transcriptomic feature selection in this study, three classical machine learning algorithms least absolute shrinkage and selection operator(LASSO) regression, support vector machine recursive feature elimination (SVM-RFE), and random forest (RF) were simultaneously applied on the basis of 20 candidate genes to precisely identify the core biomarkers. LASSO was implemented using glmnet (V 4.1-8) (Bodinier et al., 2023), which automatically optimized the penalty coefficient through 10-fold cross-validation. L1 regularization was employed to compress the coefficients of non-critical features to zero, facilitating gene screening (Narala et al., 2021); SVM-RFE was constructed using e1071 (V 1.7-13) (Lin et al., 2012), eliminating the least contributing features after each round of training, and retaining the gene subset with the strongest predictive ability.RF generates 500 decision trees using randomForest (V 4.7-1.2) (Guyon et al., 2002), quantifies feature importance through out-of-bag error evaluation and Gini gain ranking, and effectively suppresses overfitting through an ensemble voting mechanism (Hu and Szymczak, 2023). Finally, we performed a Venn intersection of the feature gene lists, selecting the overlapping genes as core candidates. Details of the three machine learning algorithms are as follows:

##### 2.3.5.1 LASSO (L1-regularized logistic regression)

In a binary (AA vs. control) logit model, incorporating L1 regularization simultaneously achieves coefficient shrinkage and variable selection (shrinking the regression coefficients of unimportant features to 0) (Tibshirani, 1996).

Model and Objective Function formula is given in Equation 1 (Binary Classification, $y_{i}\in \left\{0,1\right\}$ ):

$$\begin{array}{c} \hat{\beta }=\arg\underset{\beta \in R^{p}}{\min}\left\{-\overset{n}{\underset{i=1}{\sum }}\left[y_{i}\log\sigma \left(x_{i}^{⊤}\beta \right)+\left(1-y_{i}\right)\log\left(1-\sigma \left(x_{i}^{⊤}\beta \right)\right)\right]+\lambda \|\beta {\|}_{1}\right\},\\ \sigma \left(z\right)=\frac{1}{1+e^{-z}} \end{array}$$ (1)

where λ≥0 is a sparsity control parameter; the larger λ is, the fewer features are retained. The optimization uses coordinate descent and solves along the regularization path, and λ is selected by K -fold cross-validation (such as $\lambda_{\text{min}}$ or $\lambda_{1\mathrm{se}}$ ).

##### 2.3.5.2 SVM-RFE (support vector machine recursive feature elimination)

In linearly separable or approximately linearly separable settings, the weight vector w of linear SVM directly characterizes the contribution of each feature to the classification hyperplane; RFE iteratively eliminates the feature with the smallest weight to obtain a compact and discriminative subset (Guyon et al., 2002).

Linear SVM (Soft Margin) Objective formula is given in Equation 2:

$$\underset{w,b,\xi }{\min}\frac{1}{2}\|w{\|}_{2}^{2}+C\overset{n}{\underset{i=1}{\sum }}\xi_{i}\;\text{ s.t. }\;y_{i}\left(w^{⊤}x_{i}+b\right)\geq 1-\xi_{i},\;\xi_{i}\geq 0$$ (2)

where $y_{i}\in \left\{-1,+1\right\}$ . After training, the weight can be written as $w=\sum_{i}\alpha_{i}y_{i}x_{i}$ ( $\alpha_{i}$ is the dual variable). The RFE ranking criterion adopts the square of the per-dimensional weight shown in Equation 3:

$$\text{rank}\;\left(j\right)=w_{j}^{2}$$ (3)

In each round, several features with the smallest rank(j) are removed, and the SVM is retrained until the preset number of features is reached; the optimal subset size and penalty parameter C are selected through nested cross-validation.

##### 2.3.5.3 Random forest (RF)

RF generates an ensemble of trees through bootstrapping (bagging) and random subset feature splitting, which is suitable for nonlinear and high-order interaction scenarios; two types of importance metrics can be used to screen features (Breiman, 2001). Let the training set be ${\left\{\left(x_{i},y_{i}\right)\right\}}_{i=1}^{n}$ , where $x_{i}\in R^{p}$ is a gene expression vector (with genes as features), and $y_{i}\in \left\{0,1\right\}$ represents AA(1) vs. healthy(0). RF consists of T randomized CART trees: each tree is trained on a bootstrapped sample $S^{\left(t\right)}$ , and each node only selects the optimal split within a random subset of features (with size $m_{\text{try}}$ ); classification predictions are aggregated by voting/probability averaging presentes in Equation 4:

$${\hat{p}}_{1}\left(x\right)=\frac{1}{T}\overset{T}{\underset{t=1}{\sum }}{\hat{p}}_{t,1}\left(x\right),\;\hat{y}\left(x\right)=1\left\{{\hat{p}}_{1}\left(x\right)\geq 0.5\right\}$$ (4)

Node splitting (Gini criterion). Let the empirical class proportions at node t be $p_{k,t}\left(k\in \left\{0,1\right\}\right)$ , and the Gini impurity is given in Equation 5:

$$i_{G}\left(t\right)=1-\sum p_{k,t}^{2}$$ (5)

The impurity decrease for the candidate split $s:t\to \left\{t_{L},t_{R}\right\}$ is shown as follows Equation 6

$$\Delta i\left(s,t\right)=i_{G}\left(t\right)-\frac{n_{t_{L}}}{n_{t}}i_{G}\left(t_{L}\right)-\frac{n_{t_{R}}}{n_{t}}i_{G}\left(t_{R}\right)$$ (6)

Select the split with the largest Δi (entropy can also be used as an alternative).

Out-of-Bag (OOB) Error and Evaluation. If sample i is not drawn into the t -th tree, denote $t\in T_{i}^{\text{oob}}$ . Its OOB prediction and OOB error rate are given in Equation 7

$${\hat{y}}_{i}^{\text{oob}}=\arg\underset{k}{\max}\frac{1}{\left|T_{i}^{\text{oob}}\right|}\underset{t\in T_{i}^{\text{oob}}}{\sum }1\left\{{\hat{y}}_{t}\left(x_{i}\right)=k\right\},\;\text{ OOB}-\text{Err }=\frac{1}{n}\overset{n}{\underset{i=1}{\sum }}1\left\{{\hat{y}}_{i}^{\text{oob}}\neq y_{i}\right\}$$ (7)

For all nodes u split by gene j , accumulate the impurity decrease and weight it by the sample weight $p\left(u\right)=n_{u}/n$ of samples reaching that node; then take the average over the forest is given in Equation 8:

$$\text{MDI}\;\left(j\right)=\frac{1}{T}\overset{T}{\underset{t=1}{\sum }}\underset{\begin{array}{c} u\in t\\ \text{ split on }j \end{array}}{\sum }p\left(u\right)\Delta i\left(u\right)$$ (8)

MDI can quickly provide global rankings, but may produce bias when dealing with “different value ranges/number of categories” or highly correlated features.

First, obtain the original score $e_{\text{orig}}$ (such as accuracy/error rate) on the OOB samples. Then, randomly permute the values of the j -th gene to get $e_{\text{perm},j}$ , and define as follows Equation 9:

$${FI}_{j}=e_{\text{perm },j}-e_{\text{orig }}\;\left({FI}_{j}=\frac{e_{\text{perm },j}}{e_{\text{orig }}}-1\right)$$ (9)

the larger ${\text{FI}}_{j}$ is, the more crucial gene j is; conditional permutation can also be used to alleviate correlation bias.

#### 2.3.6 Identification of gene expression levels between normal and patient samples

We next evaluated how well the candidate feature genes distinguish AA from normal samples. We examined each feature gene’s expression in both the training and validation sets and plotted receiver operating characteristic (ROC) curves using the pROC package (v1.18.4) (Zhang et al., 2025). Genes that were differentially expressed in AA vs. controls with consistent direction in both sets and had an area-under-curve (AUC) >0.7 were deemed key diagnostic genes for subsequent analysis.

#### 2.3.7 Expression of key genes in AGA

In order to systematically investigate their expression patterns, a Comprehensive differential expression analysis of key genes was conducted in the AGA dataset. Particular emphasis was placed on the JAK family genes (JAK1, JAK2, JAK3, and TYK2), then the differential expression of these genes in AGA was analyzed to clarify their potential mechanisms in this disease.

### 2.4 Prior-informed few-shot diagnostic model

**FIGURE 1:**
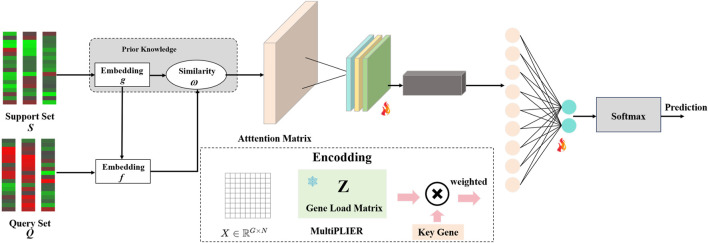
Prior-informed relation-style few-shot diagnostic model for AA.

We replaced the conventional nomogram with a Relation Network–style few-shot classifier leveraging MultiPLIER priors (Figure 1). In our model, the bulk expression matrix X (genes × samples) is first projected onto latent variables (LVs) aligned with known pathways and cell types using a fixed MultiPLIER loading matrix. The LVs corresponding to AA-relevant signals are then re-weighted based on the expression of the hub genes identified by our consensus pipeline. Next, a two-branch (support and query) encoder computes pairwise Hadamard product similarity vectors between each query and all support samples; these similarity vectors are fed into a permutation-invariant relation module and aggregated, and finally a shallow linear classifier outputs the prediction (AA or control). This design embodies the “learning to compare” concept of Relation Networks while enforcing set-level (support set) invariance. Crucially, by using a fixed biologically-informed latent space, our architecture keeps the model’s decisions transparent and mechanism-grounded–aligning with emerging XAI 2.0 principles in healthcare that demand context-rich explanations without sacrificing performance.

Step 1: Projection onto MultiPLIER Latent Space. We project the gene expression matrix X into a lower-dimensional latent space defined by a public MultiPLIER gene-by-LV loading matrix $Z\in R^{G\times L}$ (pre-trained on > 70,000 human RNA-seq samples). Specifically in formula Equation 10, we solve a ridge regression to estimate the latent variable activity matrix $B\in R^{L\times N}$ :

$$B^{*}=\arg\underset{B}{\min}\|X-ZB{\|}_{F}^{2}+\lambda \|B{\|}_{F}^{2}={\left(Z^{⊤}Z+\lambda I\right)}^{-1}Z^{⊤}X.$$ (10)

Where, Z is kept fixed (no fine-tuning) to preserve its biological alignment, which improves interpretability and prevents overfitting in small-sample settings.

Step 2: Key-gene-guided channel re-weighting.

Let $G^{*\prime }$ be the final hub-gene set from the consensus feature selection. We compute an LV-wise weight vector $\alpha \in R^{L}$ from the MultiPLIER loadings equation is given in Equation 11:

$$\alpha_{\ell }=\frac{1}{\left|G^{*\prime }\right|}\underset{g\in G^{*\prime }}{\sum }|Z_{g\ell }|,\;\tilde{B}=\text{diag}\left(\hat{\alpha }\right)\;B$$ (11)

where $\hat{\alpha }=\alpha /\max\left(\alpha \right)$ (or $\alpha /\|\alpha {\|}_{1}$ ) normalizes magnitudes. This channel gating prioritizes LVs heavily loaded by hub genes while retaining the full prior space; $\tilde{B}$ is forwarded to the encoder. (The use of a fixed prior Z keeps interpretability at the pathway/cell-type level).

Step 3: Two-Branch Encoder & Hadamard Similarity Mapping. We use a lightweight neural encoder $f_{\theta }:R^{L}\to R^{d}$ to embed each latent variable vector into a d -dimensional feature space. This encoder is shared between the support set S (AA and control reference samples) and the query sample(s). Denote the encoded embeddings as $h_{s}=f_{\theta }\left({\tilde{b}}_{s}\right)$ for each support s∈S , and $h_{q}=f_{\theta }\left({\tilde{b}}_{q}\right)$ for a query q∈Q . For a given query q , we then compute its similarity to each support sample s via the Hadamard product (element-wise multiplication) of their embeddings shown in Equation 12:

$$m_{q,s}=h_{q}⊙h_{s}\in R^{d},$$ (12)

where ⊙ denotes element-wise multiplication. This simple yet expressive operation produces a d -dimensional similarity vector $m_{q,s}$ for each query–support pair, capturing pairwise feature interactions and serving as a learnable matching mechanism in few-shot frameworks.

Step 4: Permutation-Invariant Set Aggregation. We aggregate the query–support similarity vectors across the entire support set in a permutation-invariant way (insensitive to the order of support samples). In practice, we use a Deep Sets–style relation module (The formula is shown below) that transforms and sums the similarity vectors for each class:

$$r_{q,c}=\rho_{\psi }\left(\underset{s\in S_{c}}{\sum }\phi_{\phi }\left(m_{q,s}\right)\right),\;c\in 0,1,$$

where $\phi_{\phi }$ and $\rho_{\psi }$ are small neural networks (e.g., multi-layer perceptrons or 1 × 1 convolutions). This defines a symmetric set function over the support samples and serves as a simple Relation Network–style comparator. (An attention-based Set Transformer could be used in place of the sum aggregator.) The resulting class scores $r_{q,c}$ are passed into a linear output layer with softmax to produce the final AA vs. control prediction.

Step 5: Training and Inference Procedure. We train the model in an episodic 2-way K -shot manner (two classes, K support samples per class, and a set of query samples per episode). During training, the model’s parameters are learned by minimizing the cross-entropy loss on the query predictions. For each episode, with true query label indicators $y_{q,c}$ , the loss formula is given in Equation 13:

$$L=-\underset{q\in Q}{\sum }\underset{c\in \left\{0,1\right\}}{\sum }y_{q,c}\;\log\frac{\exp\left(r_{q,c}/\tau \right)}{\sum_{c^{\prime }}\exp\left(r_{q,c^{\prime }}/\tau \right)},$$ (13)

where τ is an optional temperature hyperparameter. We apply standard $L_{2}$ weight decay and dropout regularization in the encoder $f_{\theta }$ and relation module $\left(\phi_{\phi },\rho_{\psi }\right)$ . The model is optimized using the Adam optimizer, with early stopping based on validation AUC. *Inference:* To make a prediction on a new sample, we form a support reference set from the training data and compute $r_{q,c}$ for the query; the query is then assigned to the class with the higher score $\left(\arg\max_{c}r_{q,c}\right)$ .

We used a MultiPLIER loading matrix with L = 987 latent variables. The encoder $f_{\theta }$ was a 2-layer fully connected network with 128 and 64 hidden units respectively, ReLU activation, and a dropout rate of 0.3. During training, we employed a 2-way 5-shot episodic design: each episode included 5 support samples per class (AA vs. control) and 5 query samples. The model was trained for 1,000 episodes with early stopping based on validation AUC stagnation for 10 episodes. For calibration, we compared Platt scaling and isotonic regression and adopted isotonic regression due to a lower cross-validated Brier score.

Instead of fitting a traditional logistic-regression nomogram, we trained our prior-informed few-shot model and took its raw output logit s(x) (pre-softmax score) as a risk score for AA. We then calibrated these scores to probabilistic risk estimates by learning a monotonic mapping on the training data—comparing Platt scaling (sigmoid calibration) and isotonic regression—and choosing the method with lower cross-validated Brier score. Using the selected calibration, we constructed a nomogram-style score chart: we computed the contribution of each latent feature to s(x) using SHAP (SHapley Additive exPlanation) values with respect to the post-weighting LV inputs, aggregated these contributions for each of the four hub genes via the MultiPLIER loadings, and rescaled the gene contribution totals to a 0–100 point scale. The sum of points (“Total Points”) is converted to an AA risk probability by the calibrated mapping $\hat{p}\left(x\right)=\text{Calib}\left(s\left(x\right)\right)$ . (Notably, SHAP values are additive on the log-odds scale and sum up to s(x) .) We evaluated the model’s discrimination using ROC curves (AUC) in pROC, to allow direct comparison with prior analyses, and assessed clinical utility via decision curve analysis (DCA) using the rmda package (comparing the net benefit of our model against “treat-all” and “treat-none” strategies).

### 2.5 Immune infiltration analysis

Single-sample gene set enrichment analysis (ssGSEA) was employed to quantitatively assess the abundance of 28 immune cell types between the disease group and the control group in the training set. Then the infiltration levels of the two groups were compared using the two-sided Wilcoxon rank sum test. Cell types with p < 0.05 after FDR correction were considered significantly different. To investigate potential associations between immune cell populations and the candidate key genes, he Pearson correlation coefficient between the ssGSEA score and the expression level of core genes was computed within the training set.

### 2.6 Gene set variation analysis and GSEA analyses

To delineate differences in immune response pathway activities among distinct biological groups, we first obtained 153 immune-related gene sets with complete functional annotations from the ImmPort database. Using these gene sets, we performed gene set variation analysis (GSVA) on the training set data via the R package “GSVA” (v1.46.0) to evaluate the enrichment levels of immune response pathways across samples (Hänzelmann et al., 2013). GSVA is a nonparametric algorithm that transforms a gene expression matrix into a gene set activity score matrix, enabling quantitative assessment of pathway enrichment at the single-sample level. In this way, GSVA scores reflect the enrichment status of immune-related pathways for each sample and allow comparison of pathway activities between AA and control groups.

For mechanistic insights into the roles of key hub genes in disease pathogenesis, we additionally carried out gene set enrichment analysis (GSEA) based on Hallmark gene sets. The R package “msigdbr” (v10.0.1) was employed to retrieve Hallmark reference gene sets (Sennett et al., 2024). For each hub gene, samples were stratified into high- and low-expression groups according to the median expression of that gene, and classical GSEA was applied to identify differentially regulated pathways between the two groups and to dissect the associated signaling programs (Zhang et al., 2025). GSEA evaluates whether predefined gene sets exhibit statistically significant and concordant differences between two biological states and, compared with conventional single-gene analyses, is able to detect both subtle coordinated changes across many genes and pronounced alterations within smaller gene subsets, thereby enhancing biological interpretation.

### 2.7 Identification of drug candidates

GeneCards (Stelzer et al., 2016), a comprehensive gene database integrating genomic, transcriptomic, proteomic, genetic, and functional data from approximately 150 sources, served as a key resource for exploring gene-disease associations and drug interactions. Critical gene-drug relationships based on hub genes were extracted from GeneCards, with approved drugs screened and visualized using the R package “ggalluvial” (V 0.12.5) (Brunson, 2020).

### 2.8 Single-cell sequencing analysis

Single-cell data were preprocessed and normalized using the R package “Seurat” (V 5.1.0) (Butler et al., 2018) with parameters set to min.cells = 3 and min.features = 200, retaining only genes expressed in ≥ 3 cells and cells with ≥ 200 detected genes to construct the Seurat object for subsequent analysis. Quality control filtering ensured that each cell contained 200-7,500 detected genes (nFeature), < 25,000 total RNA molecules (nCount), and < 15% mitochondrial gene content (percent.mt). Dimensionality reduction was performed via the RunPCA function, followed by cell clustering using FindNeighbors and FindClusters. Cell cluster annotation integrated multiple approaches: (1) Cluster-specific marker genes identified by FindAllMarkers were cross-referenced with the CellMarker database (Zhang et al., 2019); (2) Predictive validation was conducted using the automated single-cell annotation tool SingleR (V 2.0.0) (Aran et al., 2019); (3) Final annotations were visualized via UMAP. Expression patterns of model genes across distinct cell populations were further analyzed.

Quality control excluded cells with <200 or >7500 detected genes, total RNA count >25,000 , or mitochondrial content >15% . Doublet detection was performed using DoubletFinder, and flagged cells were removed. Batch correction across 5 AA patient samples was conducted using Seurat v5 integration with SCTransform normalization. PCA was run with 50 components, and the top 20 PCs were used for clustering (resolution =0.8 ) and UMAP embedding ( n_neighbors=30 , min_dist=0.3 ). Marker gene identification used the Wilcoxon rank-sum test with $\log_{2}\text{FC}>0.25$ and adjusted p<0.05 .

### 2.9 Multi-database functional annotation of hub genes

Cross-referencing the four hub genes and their upstream cytokines across several independent functional–annotation and signaling databases provided external validation of our computational findings. Public resources consistently annotate granzyme A (GZMA) as a cytotoxic serine protease expressed in activated ${\text{CD}8}^{+}$ T cells and natural killer cells, mediating granule-dependent target cell death and shaping inflammatory responses. Likewise, interleukin-2 (IL-2) and its receptor components are repeatedly described as central regulators of T-cell proliferation, survival, and differentiation, with established roles in driving effector and memory programs. In these databases, IL2RB and IL2RG encode the β and common γ chains shared by the IL-2/IL-15 receptor complexes, respectively, and are linked to JAK1/3 activation and downstream STAT signaling. EOMES is annotated as a T-box transcription factor that programs a cytotoxic transcriptional state in ${\text{CD}8}^{+}$ T cells, characterized by high interferon- γ and granzyme expression.

Together, this multi-database functional annotation converges on a coherent picture: the IL2RB/IL2RG–EOMES–GZMA axis represents a well-established immune effector module, and our multi-omics pipeline has rediscovered this module as the core signature distinguishing AA from AGA. This convergence increases confidence in the biological plausibility and translational relevance of the identified targets, rather than indicating a model driven by spurious associations.

### 2.10 Biological validation in independent scalp samples

#### 2.10.1 Human scalp samples and ethics

This study enrolled three groups of participants: (i) alopecia areata (AA) lesional scalp (n=10) ; (ii) androgenetic alopecia (AGA) affected scalp (n=10) ; and (iii) healthy control scalp (n=10) . The protocol was approved by the Ethics Committee of the First Affiliated Hospital of Army Medical University (approval No. ky201977). All procedures were conducted in accordance with relevant guidelines and regulations. Written informed consent was obtained from all patients/participants prior to enrollment. Scalp tissues were obtained as residual specimens from the Department of Dermatology after therapeutic excision of perilesional scalp trauma, melanocytic nevi, or lipomas. Each specimen measured approximately 0.5×0.5cm to 0.5×1.0cm . Immediately after collection, samples were snap-frozen in liquid nitrogen and stored at −80 °C until analysis.

#### 2.10.2 Western blot (WB) analysis of hub proteins

Total protein was extracted from scalp tissues homogenized on ice using RIPA lysis buffer supplemented with PMSF (Beyotime, China). Protein concentrations were determined with a BCA assay kit (Beyotime, China). Equal amounts of protein were resolved by SDS–PAGE and transferred to PVDF membranes at 200 mA for 2 h. Membranes were blocked in 5% non-fat milk at room temperature for 2 h and incubated overnight at 4 °C with primary antibodies against β -actin (1:4000; 20536-1-AP, Proteintech, China), EOMES (1:5000; 83945-5-RR, Proteintech, China), GZMA (1:500; 11288-1-AP, Proteintech, China), IL2RB (1:5000; 13602-1-AP, Proteintech, China), and IL2RG (1:500; 11409-1-AP, Proteintech, China). After TBST washes, membranes were incubated with HRP-conjugated secondary antibodies (Beyotime, China) for 2 h at room temperature and washed again with TBST. Signals were visualized by enhanced chemiluminescence.

#### 2.10.3 Real-time quantitative polymerase chain reaction (RT-qPCR) analysis of hub genes

Total RNA was isolated using the RNASimple Total RNA Kit (TIANGEN, China). First-strand cDNA was synthesized with the MightyScript First-Strand cDNA Synthesis Mix (Sangon, China). qPCR was performed with 2 × SG Fast qPCR Master Mix (Sangon, China) on a Bio-Rad CFX96 real-time PCR system (Bio-Rad, United States) under the following conditions: initial denaturation at 95 °C for 3 min; 40 cycles of 95 °C for 3 s and 60 °C for 20 s for annealing/extension/data acquisition. Primer sequences are listed in Table 1.

**TABLE 1:** The sequences of primers used for qRT-PCR detection.

Gene	Primer (5′–3′)	Sequence
EOMES	Forward	CAATCCTTCTTCCCGGAGCC
	Reverse	GTTTGTTGGTCCCAGGTTGC
GZMA	Forward	GGAACCATGTGCCAAGTTGC
	Reverse	CACGAGTCTCTTCCACCTCG
IL2RB	Forward	TTGGGAAGGACACCATTCCG
	Reverse	GACGTCTCCTCCATGCTCTG
IL2RG	Forward	TGGATGGGCAGAAACGCTAC
	Reverse	ATAACCACGGCTTCCAATGC
β -Actin	Forward	ACCCCGCCGCCAGCTCACC
	Reverse	GGGGGGCACGAAGGCTCATC

## 3 Result

**FIGURE 2:**
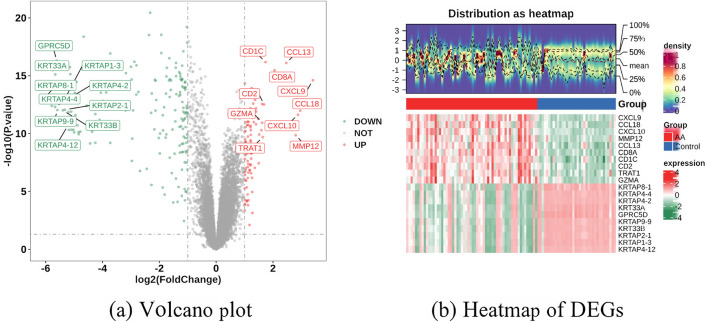
Screening of differentially expressed genes (DEGs) and identification of module genes in alopecia areata (AA). **(a)** Volcano plot and **(b)** Heatmap of DEGs across different groups (Control and AA).

During differential expression analysis of the AA training set, a total of 257 differentially expressed genes (DEGs) were identified. Among these, 91 DEGs exhibited upregulated expression, while 166 DEGs showed downregulated expression. Based on these results, a volcano plot was generated to visualize the changes in gene expression, as shown in Figure 2a. Furthermore, the top 10 most DEGs from both the upregulated and downregulated groups were extracted, and a heatmap was plotted to examine their distribution patterns across samples, as shown in Figure 2b. Additionally, WGCNA was performed using the training set data, with disease status encoded as the phenotypic trait.

**FIGURE 3:**
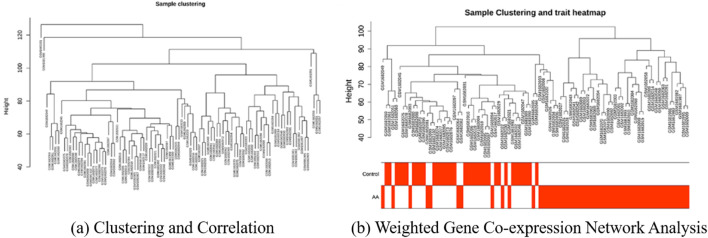
Screening of differentially expressed genes (DEGs) and identification of module genes in alopecia areata (AA). **(a)** Sample clustering tree diagram and correlation heatmap and **(b)** Scale independence and average connectivity of different soft threshold powers used in Weighted Gene Co-expression Network Analysis (WGCNA).

**FIGURE 4:**
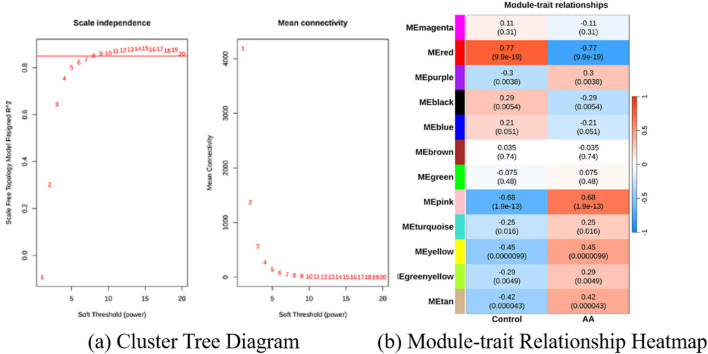
Screening of differentially expressed genes (DEGs) and identification of module genes in alopecia areata (AA). **(a)** Cluster tree diagram generated by WGCNA and **(b)** Module-trait relationship heatmap showing the correlation coefficients and significance values between modules and traits of interest.

During this process, six outlier samples (GSM1681988, GSM1681991, GSM1682055, GSM1682056, GSM1682057, and GSM1682101) were identified and excluded to ensure the accuracy and reliability of the analysis, as shown in Figure 3a. By setting a scale-free network assessment coefficient ( $R^{2}$ = 0.85) and an optimal soft threshold ( β = 8), 12 distinct gene co-expression modules were successfully identified, as shown in Figures 3b, 4. Subsequently, based on a Pearson correlation threshold (p < 0.05; | cor |> 0.6), the modules exhibiting the most significant positive (red module) and negative (pink module) correlations with phenotypic traits were selected, from which 1,667 associated genes were determined, as shown in Figure 4b. These genes provide profound basic data for subsequent research.

**FIGURE 5:**
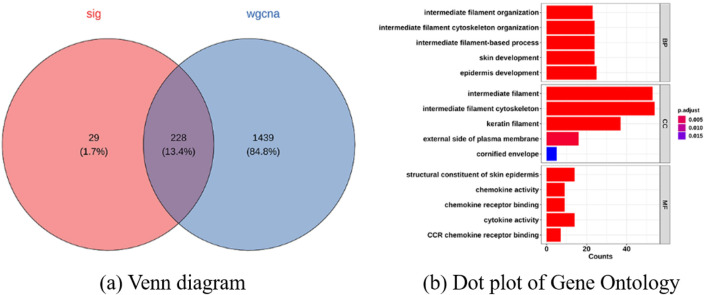
Functional enrichment and gene expression analysis. **(a)** Venn diagram showing the overlap between genes identified by DEGs and WGCNA and **(b)** Dot plot of Gene Ontology (GO) terms for cellular component (CC), biological process (BP), and molecular function (MF) enriched among DEGs.

By intersecting the DEGs with genes from the significant WGCNA modules, we obtained 228 overlapping genes (Figure 5a). We performed GO enrichment for these genes and visualized the top five categories in each GO domain (Figure 5b).

**FIGURE 6:**
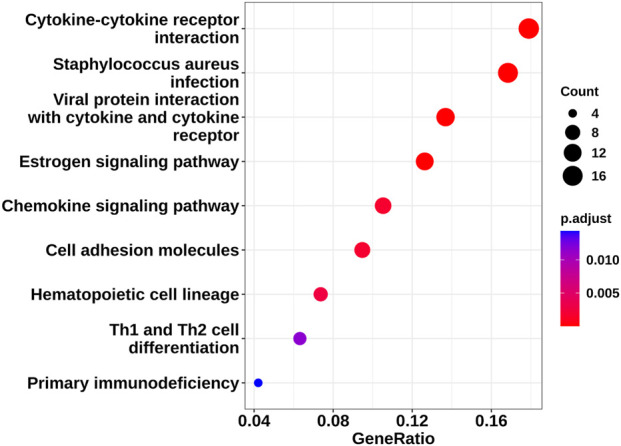
Dot plot of KEGG enrichment analysis pathway.

For Biological Process (BP), the most enriched terms were intermediate filament organization, intermediate filament-based process, skin development, and epidermal development. For Cellular Component (CC), top terms included molting cycle, intermediate filaments (and intermediate filament cytoskeleton), keratin filaments, and external side of plasma membrane. For Molecular Function (MF), top terms included keratinization, structural constituent of skin epidermis, chemoattractant activity, chemoattractant receptor binding, cytokine activity, and CCR chemokine receptor binding. Additionally, KEGG pathway analysis identified nine significantly enriched pathways (Figure 6), including *Staphylococcus aureus* infection, viral protein–cytokine/chemokine interactions, cytokine–cytokine receptor interaction, estrogen signaling, chemokine signaling, cell adhesion molecules (CAMs), hematopoietic cell lineage, Th1 and Th2 cell differentiation, and primary immunodeficiency. These enrichment results provide a context-sensitive interpretation of the overlapping genes, suggesting they cluster in pathways relevant to AA pathology.

**FIGURE 7:**
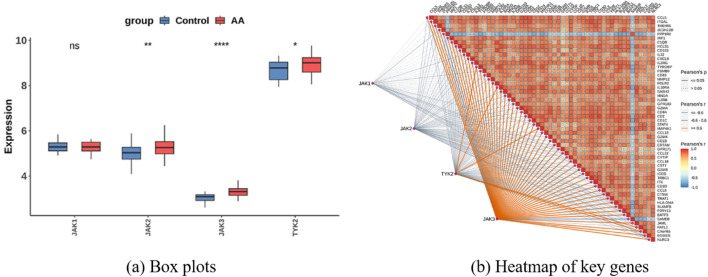
Functional enrichment and gene expression analysis. **(a)** Box plots comparing the expression levels of Janus kinase (JAK) family genes (JAK1, JAK2, JAK3, TYK2) between control and AA groups and **(b)** Heatmap of the correlation matrix of key genes, with Pearson’s correlation coefficients shown. Nodes and edges highlight the relationships among JAK family genes and others.

First, Pearson correlation analysis was used to pair all DEGs with the expression levels of JAK1, JAK2, JAK3, and TYK2 one by one and calculate the correlation coefficients. Subsequently, genes with statistically significant correlations with JAK family genes were screened for further study. The results showed 55 genes were positively or negatively correlated with at least one member of the JAK axis at a moderate-intensity level. Thus, they were included in the candidate list for subsequent functional verification. The correlation results are presented as a heatmap: colors range from light to dark, corresponding to correlation coefficients from weak to strong, and symbols in the squares indicate statistical significance, as shown in Figure 7.

**FIGURE 8:**
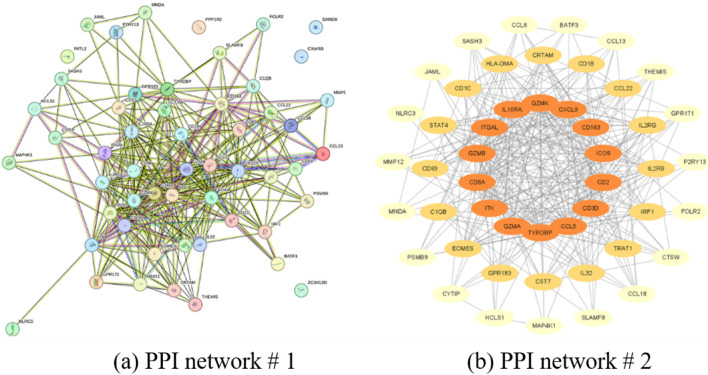
Identification hub genes in AA. **(a,b)** PPI network showing the complex interactions between key genes.

**FIGURE 9:**
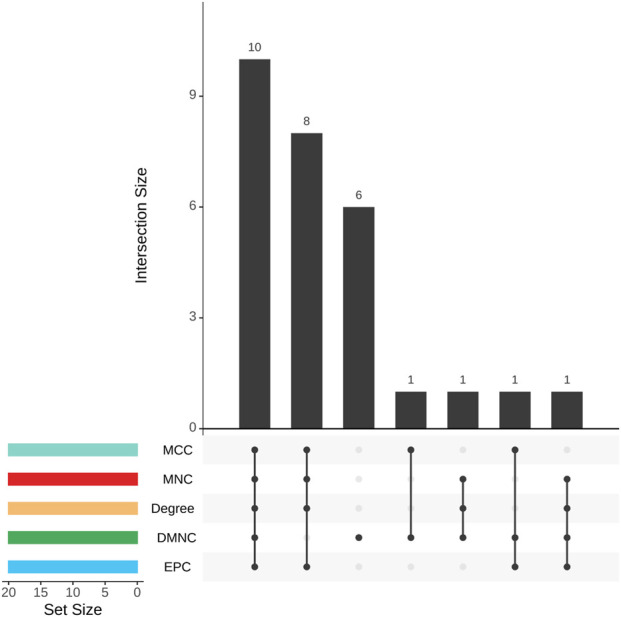
Upset plot of 10 intersecting genes obtained from five topology analysis algorithms (Maximum Clique Centrality (MCC), Maximum Neighborhood Component (MNC), Degree, Density of Maximum Neighborhood Component (DMNC), Edge Percolated Component (EPC)) in the cytoHubba plugin.

Genes found to be strongly correlated with the JAK family underwent PPI network analysis utilizing STRING, with visualization achieved through Cytoscape. This network-based approach facilitated the identification of 10 core hub genes: cluster of differentiation 3D (CD3D), granzyme A (GZMA), interleukin 2 receptor subunit beta (IL2RB), interleukin 2 receptor subunit gamma (IL2RG), inducible T-cell costimulator (ICOS), STAT4, interleukin 10 receptor subunit alpha (IL10RA), eomesodermin (EOMES), interleukin-2-inducible T-cell kinase (ITK), and integrin subunit alpha L (ITGAL), as shown in Figures 8, 9.

**FIGURE 10:**
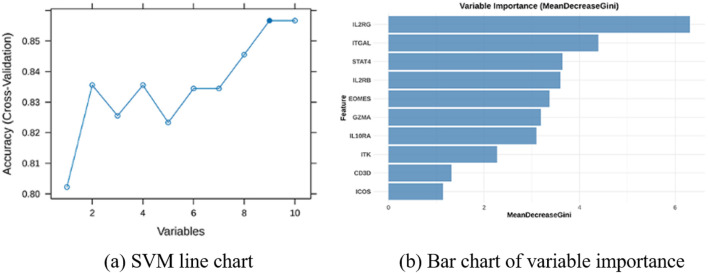
Identification hub genes in AA. **(a)** The Support Vector Machine (SVM) line chart shows that when the model reaches its highest accuracy, nine genes are selected. **(b)** Bar chart of variable importance based on MeanDecreaseGini from Random Forest analysis, illustrating the most critical genes.

**FIGURE 11:**
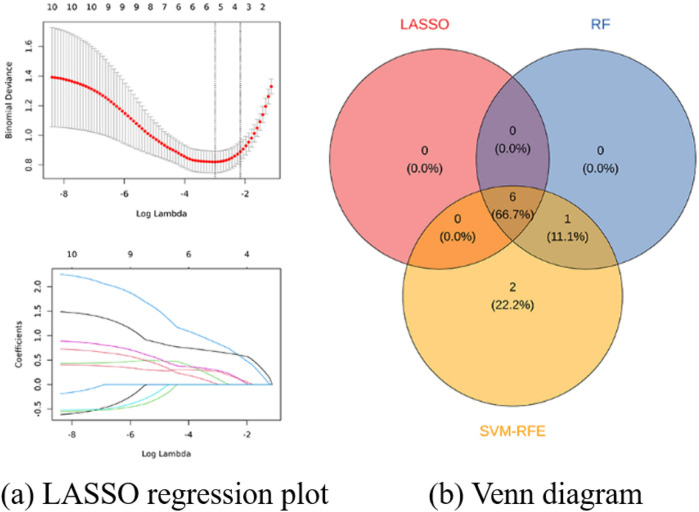
Identification hub genes in AA. **(a)** Least absolute shrinkage and selection operator (LASSO) regression plot displaying binomial deviance versus log lambda values to determine optimal gene selection. **(b)** Venn diagram representing the overlap of identified genes from LASSO, Random Forest (RF), and SVM-Recursive Feature Elimination (RFE).

To rigorously refine the selection of key molecular drivers, three complementary machine learning algorithms were implemented on the training dataset. The LASSO regression model first highlighted six pivotal genes, namely, GZMA, IL2RB, IL2RG, STAT4, IL10RA, and EOMES, as shown in Figure 11a. Following this, SVM-RFE analysis delineated nine influential genes, including IL2RG, IL10RA, GZMA, ITGAL, CD3D, STAT4, EOMES, IL2RB, and ITK, as shown in Figure 10a. dditionally, the RF classifier, applying a MeanDecreaseGini threshold exceeding 3, pinpointed seven key genes: IL2RG, ITGAL, STAT4, IL2RB, EOMES, GZMA, and IL10RA, as shown in Figure 10b. By intersecting the outputs derived from these three distinct analytical frameworks, a robust consensus set of six feature genes: GZMA, IL2RB, IL2RG, STAT4, IL10RA, and EOMES was ultimately determined, as shown in Figure 11b. This integrative approach reinforced the credibility of the selected genes as pivotal components within the JAK-related molecular landscape.

**FIGURE 12:**
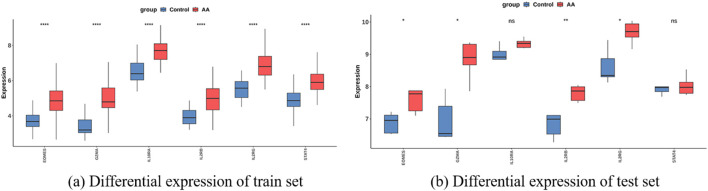
Validation and predictive performance of feature genes in AA. **(a)** Differential expression of six core genes in the AA and control groups of the training set. **(b)** Differential expression of six core genes in the AA and control groups of the test set.

**FIGURE 13:**
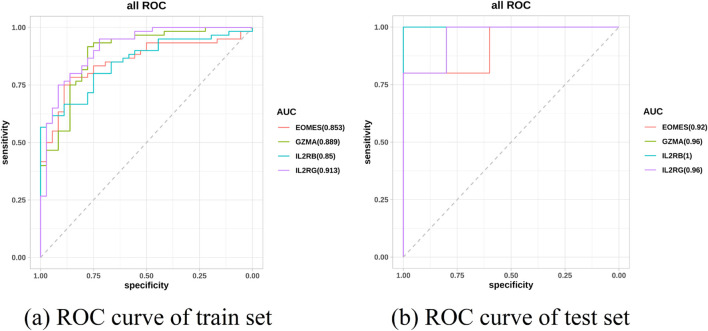
Validation and predictive performance of feature genes in AA. **(a)** Receiver operating characteristic (ROC) curve of six core genes in the AA and control groups of the training set. **(b)** ROC curve of six core genes in the AA and control groups of the test set.

**FIGURE 14:**
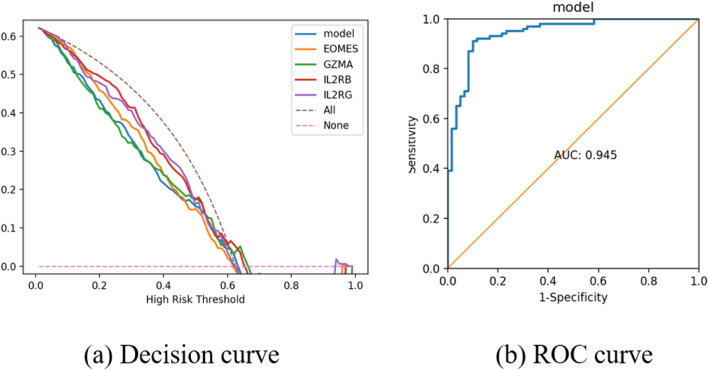
**(a)** Decision curve analysis illustrating the net benefit of the predictive model across different risk thresholds. **(b)** ROC curve for the model, with the area under the curve (AUC) indicating the model’s overall predictive power.

Based on these six feature genes, an external validation cohort was further introduced for expression validation. Based on these six feature genes, an external validation cohort was further introduced for expression validation. Of the six candidate biomarkers identified by our machine-learning selection, four genes—GZMA, IL2RB, IL2RG, and EOMES—showed significant differential expression in an independent AA cohort, with consistent up/downregulation trends (Figure 12). Each of these four genes achieved an AUC >0.70 for distinguishing AA from controls (Figure 13).We therefore designated these four as the core biomarkers for our diagnostic model. Using the training set, we trained the few-shot model on these biomarkers and evaluated its performance. The model achieved high discriminatory power (AUC =0.945 ) as shown by ROC analysis, and decision curve analysis (Figure 14) indicated improved clinical net benefit of the model across a range of risk thresholds (outperforming “treat-all” and “treat-none” strategies).

**FIGURE 15:**
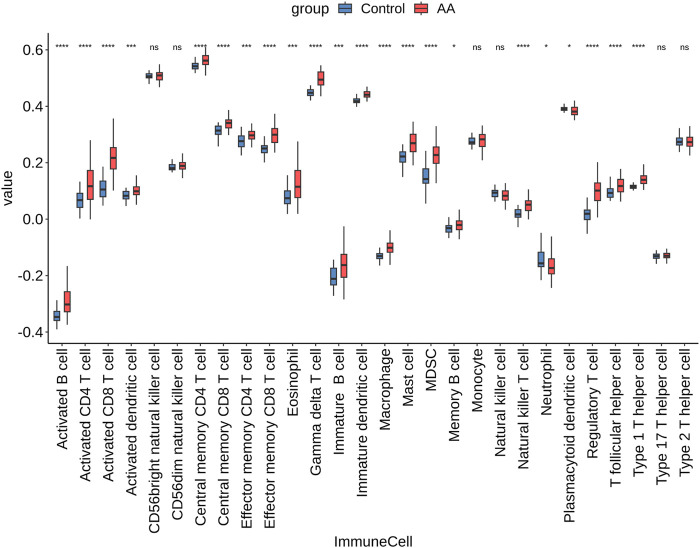
Immune cell infiltration analysis. Box plots comparing the infiltration levels of 28 immune cell types between the control and AA groups.

**FIGURE 16:**
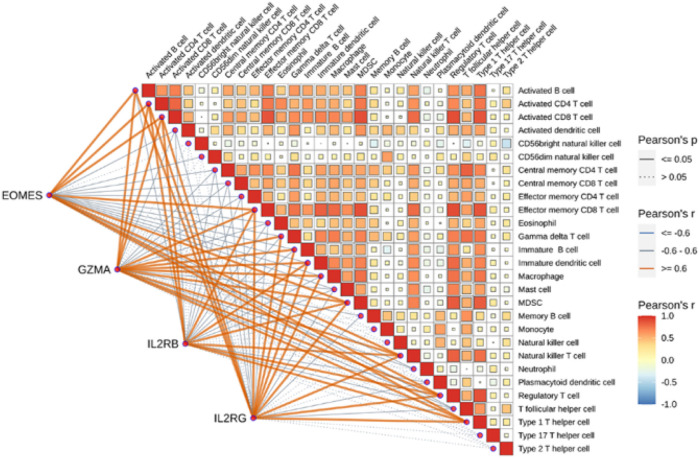
Immune cell infiltration analysis. Correlation heatmap showing Pearson’s correlation coefficients between the infiltration levels of different immune cell types and hub genes. Orange lines denote significant positive correlations, while blue lines indicate significant negative correlations.

We analyzed immune cell infiltration in AA lesions versus controls. Out of 28 cell types, 22 showed significantly different abundance between AA and healthy scalps ( p<0.05 , Figure 15). In particular, AA lesions had elevated levels of multiple T cell subsets (e.g., activated ${\text{CD}4}^{+}$ T, activated ${\text{CD}8}^{+}$ T, effector memory T, regulatory T cells) and other immune cells (e.g., activated B cells, dendritic cells, macrophages, NK cells, MDSCs), compared to controls. Furthermore, the four hub genes were each strongly positively correlated (Pearson r , p<0.05 ) with a subset of these infiltrating immune cells. For instance, higher GZMA expression correlated with increased activated ${\text{CD}8}^{+}$ T cells and effector memory ${\text{CD}8}^{+}$ T cells; IL2RB and IL2RG levels correlated with T cell and NK cell subsets; and EOMES correlated with cytotoxic T cell levels (Figure 16). These findings suggest that AA is characterized by a broad immune cell infiltration, tightly linked to the identified hub genes.

**FIGURE 17:**
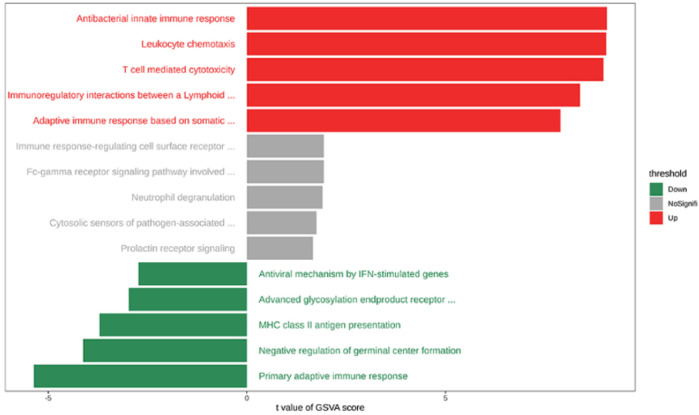
Bar plot showing the differential analysis results of gene set variation analysis (GSVA).

Gene set variation analysis (GSVA) of the training set revealed widespread immune pathway perturbations in AA. We identified 115 immune-related pathways with significant activity differences relative to controls (p<0.05) : 109 pathways showed higher activity in AA, while 6 were lower. Figure 17 visualizes the five most extreme pathways in each category (upregulated, downregulated, and unchanged). Notably, AA lesions showed elevated activity in pathways related to innate and adaptive immunity—e.g., antibacterial innate immune response, leukocyte chemotaxis, T cell–mediated cytotoxicity, immune interactions between lymphoid and non-lymphoid cells, and adaptive immune responses involving antigen receptor recombination. In contrast, a few pathways were suppressed in AA, such as primary adaptive immune response, negative regulation of germinal center formation, MHC class II antigen presentation, advanced glycation end-product receptor signaling, and antiviral interferon-stimulated gene pathways. GSEA was performed using the HALLMARK gene set as the reference.

Together, these concordant annotations converge on a coherent picture: the IL2RB/IL2RG–EOMES–GZMA axis represents a well-established immune effector module rather than a spurious statistical pattern, and our multi-omics pipeline has rediscovered this module as the core signature distinguishing AA from AGA. This convergence increases confidence in the biological plausibility and translational relevance of the identified targets and supports their prioritization for pathway-guided immunomodulatory therapies.

**FIGURE 18:**
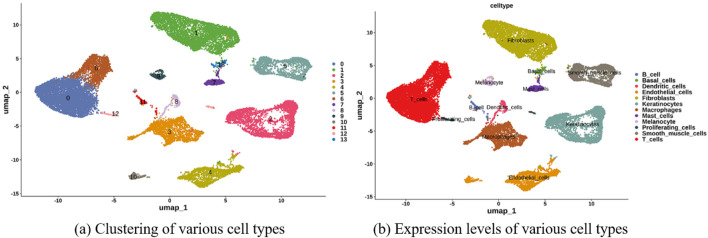
Single-cell analysis. **(a)** Uniform Manifold Approximation and Projection (UMAP) displaying the clustering of various cell types. **(b)** Dot plot illustrating the expression levels of distinct marker genes across identified cell types.

**FIGURE 19:**
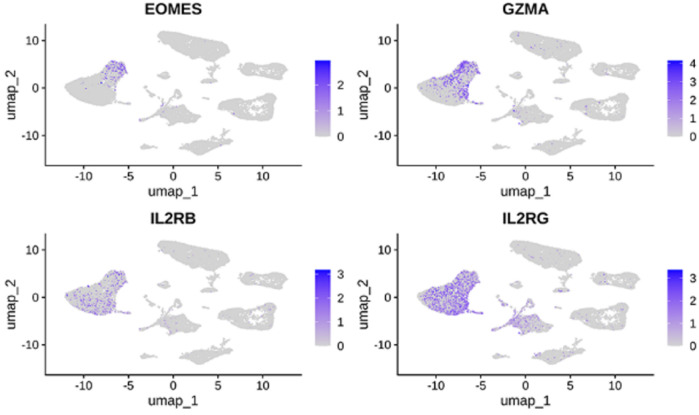
UMAP feature plots showing the expression of hub genes across different cell clusters.

**FIGURE 20:**
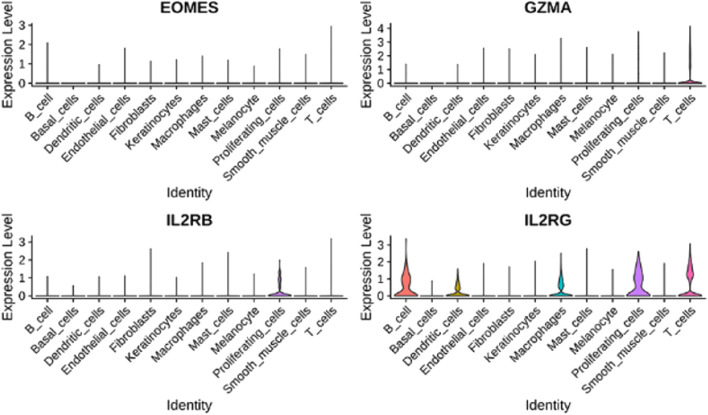
Violin plots depicting the distribution and expression levels of hub genes in each cell type.

Using single-cell RNA-seq from AA lesions (5 samples, 25,875 cells), we identified 12 distinct cell clusters (Figure 18). The hub genes were predominantly expressed in immune cell populations. In AA, GZMA and EOMES were enriched in ${\text{CD}8}^{+}$ T cells, and IL2RB and IL2RG were notably expressed in proliferating T cells and other lymphocytes (Figures 19, 20).

**FIGURE 21:**
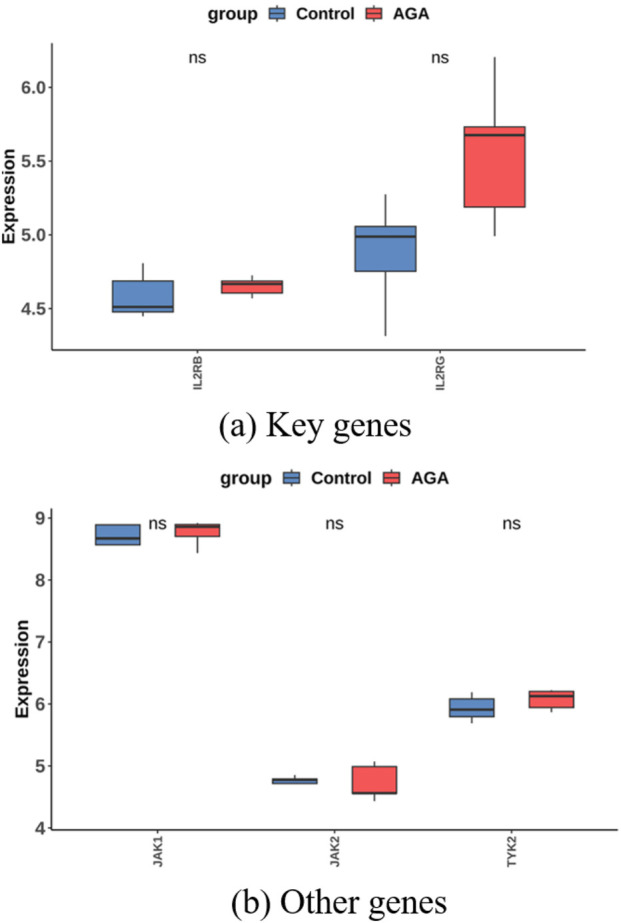
The expression of key genes and JAK family in AGA. **(a)** Box plots showing the expression levels of key genes between the Control and AGA groups (EOMES and GZMA were not exist in the AGA dataset). **(b)** Box plots depicting the expression levels of JAK1, JAK2, and TYK2 genes in control versus AGA groups (JAK3 transcripts were not detected in the AGA dataset).

These findings outline a localized immune circuit in AA lesions: the hub genes are active in cytotoxic T cells and proliferating lymphocytes, suggesting a central cytotoxic T-cell loop driving AA pathology. By contrast, in AGA scalps the expression of these hub genes was minimal or unchanged (Figure 21). Neither EOMES nor GZMA was detected in the AGA single-cell data, and JAK–STAT pathway genes (JAK1/2/3, TYK2) showed no significant up- or downregulation. Thus, unlike AA, AGA did not exhibit a notable JAK–STAT immune activation signature at the single-cell level.

**FIGURE 22:**
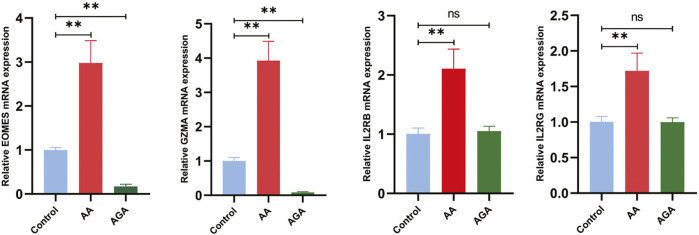
RT-qPCR validation of the IL2RB/IL2RG–EOMES–GZMA cytotoxic/JAK axis in alopecia areata (AA). Relative mRNA levels of EOMES, GZMA, IL2RB, and IL2RG are significantly elevated in AA lesional scalp compared with healthy controls, whereas androgenetic alopecia (AGA) samples are comparable to controls, indicating that this cytotoxic/JAK module is transcriptionally active in AA but largely quiescent in AGA. Data are shown as mean ± SD. Statistical annotations: ** p<0.01 vs. control; n.s., not significant (AGA vs. control).

To determine whether this cytotoxic/JAK axis is recapitulated in independent patient tissues, we next examined its expression in AA, AGA, and healthy scalp specimens. Compared with healthy controls, mRNA levels of EOMES, GZMA, IL2RB, and IL2RG were significantly upregulated in alopecia areata (AA) lesional scalp (all p<0.01 ), whereas androgenetic alopecia (AGA) scalp showed no significant increase relative to controls (all p>0.05 ). In AA, the transcription factor EOMES and the cytotoxic effector GZMA exhibited the most pronounced elevations; IL2RB/IL2RG were also consistently increased, indicating activation of the IL-2/IL-15–JAK1/3 axis (Figure 22). These AA-specific mRNA elevations are fully consistent with our microarray-based differential expression, external validation cohorts, and immune-infiltration analyses, and they further underscore that the IL2RB/IL2RG–EOMES–GZMA axis is transcriptionally active in AA but quiescent in AGA.

## 4 Discussion

The infiltration levels of 28 immune cell types in the training set were analyzed using the ssGSEA algorithm to compare differences between the disease group and the control group. It is important to note that the AGA dataset (GSE36169) comprised only five AGA samples and five controls, which substantially limits statistical power. This small sample size may lead to an underestimation of subtle AGA-specific transcriptomic changes, including immune or inflammatory signals that fall below our detection threshold. Accordingly, our conclusion that AGA lacks a comparable inflammatory JAK–STAT signature should be interpreted with caution and validated in larger, independent cohorts.

**FIGURE 23:**
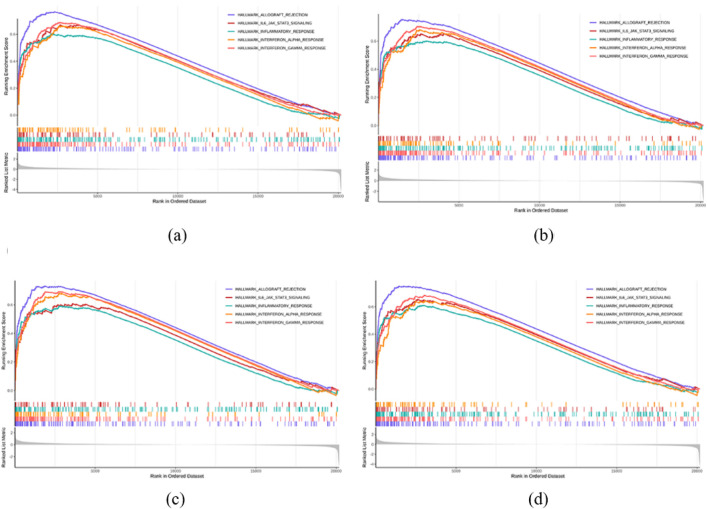
GSEA plots for key genes. **(a)** EOMES. **(b)** GZMA. **(c)** IL2RB. **(d)** IL2RG.

A significance threshold of normalized enrichment score (NES) > 1 and p < 0.05 was applied, and the top five significantly enriched pathways were visualized. We also performed single-gene gene set enrichment analysis (GSEA) for each hub gene using Hallmark gene sets. The top enriched pathways linked to the hub genes included graft rejection, IL6–JAK–STAT3 signaling, inflammatory response, interferon alpha response, and interferon gamma response (Figure 23). These enrichment patterns further underscore the central role of JAK–STAT signaling and broad immune activation in AA’s gene expression profile.

**FIGURE 24:**
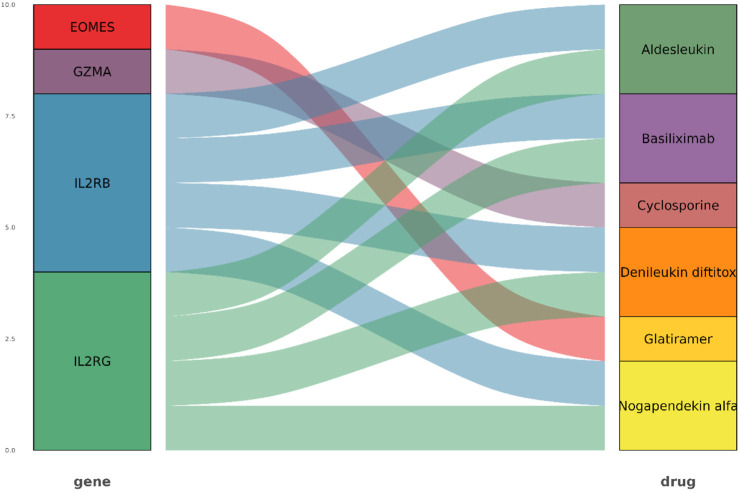
Sankey diagram of hub genes and corresponding drugs.

Four key genes identified as related to drugs was selected from the GeneCards database, their connections to therapeutic drugs were visualized using a Sankey diagram, as shown in Figure 24. The EOMES gene was found to have a significant association with glatiramer, while GZMA was closely linked to cyclosporine. In addition, IL2RB and IL2RG were found to correlate with four clinically significant drugs, namely, Basiliximab, Denileukin diftitox, Aldesleukin, and Nogapendekin alfa (Anktiva). In summary, these drugs play an important role in clinical practice, highlighting their therapeutic significance.

Beyond gene-level associations and drug–gene networks, we next sought to determine whether the key components of this cytotoxic/JAK axis are consistently annotated as immune effector molecules in independent functional-annotation resources. Cross-referencing the four hub genes and their upstream cytokines across several databases provided external validation of our computational findings. Public resources consistently annotate granzyme A (GZMA) as a cytotoxic serine protease expressed in activated ${\text{CD}8}^{+}$ T cells and natural killer cells, mediating granule-dependent target cell death and shaping inflammatory responses. Likewise, interleukin-2 (IL-2) and its receptor components are repeatedly described as central regulators of T-cell proliferation, survival, and differentiation, with established roles in driving effector and memory programs. In these databases, IL2RB and IL2RG encode the β and common γ chains shared by the IL-2/IL-15 receptor complexes, respectively, and are linked to JAK1/3 activation and downstream STAT signaling. EOMES is annotated as a T-box transcription factor that programs a cytotoxic transcriptional state in ${\text{CD}8}^{+}$ T cells, characterized by high interferon- γ and granzyme expression.

**FIGURE 25:**
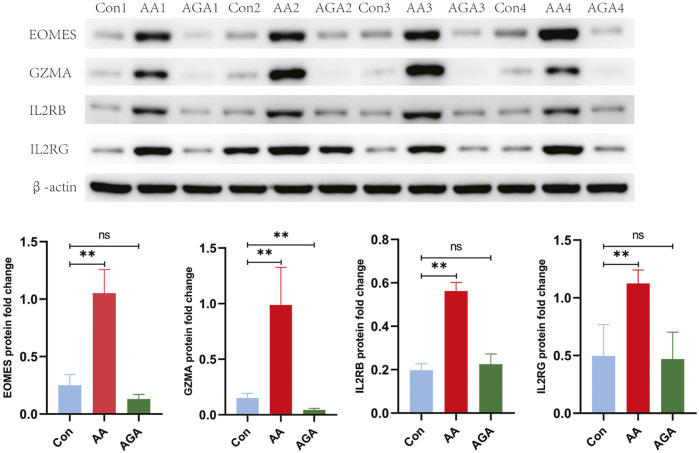
Western blot validation of the IL2RB/IL2RG–EOMES–GZMA cytotoxic/JAK axis at the protein level. Protein expression of EOMES, GZMA, IL2RB, and IL2RG is markedly upregulated in AA lesional scalp compared with healthy controls, concordant with the RT-qPCR results, whereas AGA-affected scalp shows no significant difference relative to controls. Representative immunoblots and densitometric quantification (normalized to β -actin) are shown. Data are presented as mean ± SD. Statistical annotations: ** p<0.01 vs. control; n.s., not significant (AGA vs. control).

Protein expression changes paralleled the qPCR results: AA samples displayed markedly higher levels of EOMES, GZMA, IL2RB, and IL2RG proteins than healthy controls (p<0.01) , while AGA did not differ significantly from controls. In AGA, none of the targets exceeded control levels; in some cohorts, EOMES/GZMA signals approached or fell below the detection threshold (Figure 25), arguing against an AA-like cytotoxic–JAK loop in AGA. Taken together, the concordant mRNA and protein-level changes in AA, contrasted with the flat expression profiles in AGA, provide orthogonal wet-lab validation that the cytotoxic/JAK axis identified *in silico* is a genuine, disease-specific effector module rather than a computational artifact.

Despite both causing hair loss, AA and AGA are driven by fundamentally different molecular mechanisms. Our multi-omics analysis, single-cell analysis, and wet-lab validation together delineated a clear split: AA lesions are transcriptionally co-dominated by hyperactive JAK–STAT signaling and a granzyme-rich cytotoxic ${\text{CD}8}^{+}$ T-cell infiltrate, whereas AGA lesions retain an androgen-centric, low-inflammation profile with minimal JAK–STAT engagement. This dichotomy aligns with clinical observations—AA is a cytotoxic T-cell–driven disease reversible by JAK inhibitors, while AGA is not—and it provides a unified framework to tailor mechanism-based therapies. Building on these insights, we pinpointed four hub genes in AA (GZMA, IL2RB, IL2RG, EOMES) using network analysis and machine learning, and confirmed their importance in independent cohorts. Notably, EOMES, a transcription factor guiding ${\text{CD}8}^{+}$ T-cell effector/memory differentiation, links the presence of perifollicular ${\text{EOMES}}^{+}$ ${\text{CD}8}^{+}$ T cells in AA to the observed cytotoxic immune phenotype. RT-qPCR and Western blot analyses in independent AA/AGA/healthy scalp samples confirmed that EOMES, GZMA, IL2RB and IL2RG are robustly upregulated at both mRNA and protein levels in AA but not in AGA, mirroring the transcriptomic and single-cell patterns.

Unlike the conventional logistic nomogram, our diagnostic model uses a prior-informed few-shot learning approach. It first embeds each sample’s expression into pathway/cell-type–aligned latent variables via fixed MultiPLIER loadings (ensuring portability and interpretability in small-sample settings). It then performs a full support–query comparison using Hadamard-product similarity and permutation-invariant aggregation to learn a class relation score (“learning to compare”), and finally outputs the AA vs. control prediction through a shallow classifier. Our framework embodies three key advantages: (1) By leveraging a pathway-aligned latent space (MultiPLIER priors), the model remains robust in small-sample settings and its features have direct biological meaning, enhancing interpretability. (2) The few-shot, relation-based approach (“learning to compare”) is well-suited to situations with limited cohorts yet a need to generalize, aligning with Healthcare 4.0’s emphasis on data-efficient AI. (3) The model’s risk output is easily translated into a familiar nomogram-style score (after calibration), with each gene’s contribution explained via additive SHAP values. This transparency ensures the predictions are clinically interpretable, facilitating trust and communication between AI, clinicians, and patients. Collectively, these features illustrate a paradigm of XAI 2.0: our framework balances high accuracy with transparent, context-sensitive reasoning, thereby fostering greater trust in AI-driven clinical decisions. Additionally, we evaluated our model with ROC curves and decision curve analysis to facilitate fair comparison with traditional prediction models, and it demonstrated superior performance and net benefit under these metrics.

Multi-perspective explanations for different stakeholders: Our framework’s interpretability can be tailored to various end-users in the clinical setting. For clinicians, the nomogram-style scorecard provides a transparent decision aid, linking gene expression to risk on an intuitive 0–100 scale. For patients, results can be translated into clear terms (e.g., “high immune activity gene levels indicate active disease, suggesting JAK inhibitor therapy may be effective”), enhancing understanding and shared decision-making. For research scientists, the model’s latent features and SHAP attributions supply mechanistic insights by mapping gene contributions to pathways and cell types. Notably, the MultiPLIER latent variables with the highest re-weighting coefficients correspond to the key pathways identified in AA: for example, one top latent factor is annotated with a cytotoxic T-cell signature (enriched in genes such as *GZMA* and *EOMES*), and another is associated with cytokine/JAK–STAT signaling (enriched in *IL2RB*, *IL2RG*, etc.). These highly weighted latent dimensions align with the JAK–STAT hyperactivation and cytotoxic T-cell activity driving AA, demonstrating that our model’s predictions are grounded in biologically interpretable features. This multi-role adaptability ensures the AI’s explanations are context-sensitive—providing the right depth of information to the right audience—thereby building trust and accountability in a Healthcare 4.0 environment.

At the mechanistic level, our findings identify JAK-STAT activity and cytotoxic T cell signaling as the two pathways most enriched in AA, positioning them within the same EOMES^+^ CD8^+^ cell cluster. This supports the functional axis “JAK-STAT → cytotoxic T cells” as the pathological core of AA. This unified model explains the reversal effect of broad-spectrum JAK inhibitors in AA, clarifies the limited efficacy of single-cytokine therapies, and provides rationale for next-generation combination interventions combining “JAK blockade + direct regulation of cytotoxic T cell function.” Notably, baricitinib (2022) was approved in 2022 for severe adult AA, followed by oral JAK inhibitors like ritlecitinib (2023), further clinically validating this pathway’s pathogenic role. The four pivotal genes exhibit distinct roles within this axis: IL2RB/IL2RG promotes clonal expansion and maintains effector programs via JAK1/3 coupling; EOMES locks cells into a cytotoxic transcriptional state characterized by high IFN- γ /high granzyme expression; GZMA executes effector killing. Consistent with multi-database annotations cataloguing IL-2 and granzyme A as canonical immunomodulatory and cytotoxic mediators, this IL2RB/IL2RG–EOMES–GZMA circuit represents not only a transcriptional signature but a biologically grounded effector axis with established roles in T-cell activation and target-cell damage. Pathway enrichment and protein interaction networks situate them within broader immune programs (cytokine receptor interactions, Th1 differentiation, granzyme-mediated cytotoxicity, etc.), suggesting synergistic effects at both upstream amplification and downstream effector levels beyond follicular attack. Integrating public drug perturbation resources, this “IL2RB/IL2RG-EOMES-GZMA” circuit shows the strongest “inverse” correlation with JAK inhibitors at the transcriptional level, providing a basis for prioritizing evaluation of strategies such as IL-2 pathway modulation and granzyme targeting. In essence, by leveraging domain-specific knowledge, our model not only predicts outcomes but also yields mechanistic explanations that clinicians can readily interpret—exemplifying the value of domain-grounded explainability in XAI 2.0 for healthcare.

## 5 Conclusion

In summary, our multi-layered analyses identified a four-gene cytotoxic loop (GZMA, IL2RB, IL2RG, EOMES) driving pathological JAK–STAT hyperactivation in AA, whereas AGA remains governed by androgenic signaling with minimal JAK–STAT involvement. Multi-database functional annotation and independent RT-qPCR and Western blot assays convergently confirmed that this IL2RB/IL2RG–EOMES–GZMA axis is selectively activated in AA at both the transcript and protein levels, providing orthogonal support for its use as a mechanistic biomarker and therapeutic target. This mechanistic split sharpens the molecular boundary between AA and AGA and maps naturally onto therapeutic strategy: AA benefits from pathway-directed immunomodulation, now clinically validated with JAK inhibitors such as baricitinib and ritlecitinib, and suggests upstream (IL-2 receptor) or downstream (cytotoxic effector) nodes as complementary targets; AGA, by contrast, remains best addressed with androgen suppression (finasteride) and minoxidil, with Wnt/ β -catenin modulation representing an active investigational avenue. Beyond these biological insights, our prior-informed few-shot classifier offers a practical, explainable diagnostic tool that leverages pathway- and cell-type–aware features, making it well-suited for small-cohort precision medicine. This approach can facilitate biomarker-guided, personalized management of hair loss disorders by providing robust and transparent predictions even when sample sizes are limited. In summary, integrating biological priors with few-shot learning may serve as a generalizable blueprint for other transcriptomic diagnostic applications facing small sample constraints. This work exemplifies how coupling domain knowledge with few-shot learning advanced XAI 2.0 in Healthcare 4.0, paving the way for trustworthy, context-sensitive AI diagnostics in future precision medicine.

## Data Availability

The datasets presented in this study can be found in online repositories. The names of the repository/repositories and accession number(s) can be found in the article/Supplementary Material.
