# Supplementary figures and images for: JAK-centric explainable few-shot gene-expression diagnosis framework for alopecia via MultiPLIER priors and relation-style set-to-set comparison
Source: Front Mol Biosci. 2026 Jan 12;12:1753206. doi: 10.3389/fmolb.2025.1753206 (PMC12832304; doi:10.3389/fmolb.2025.1753206)

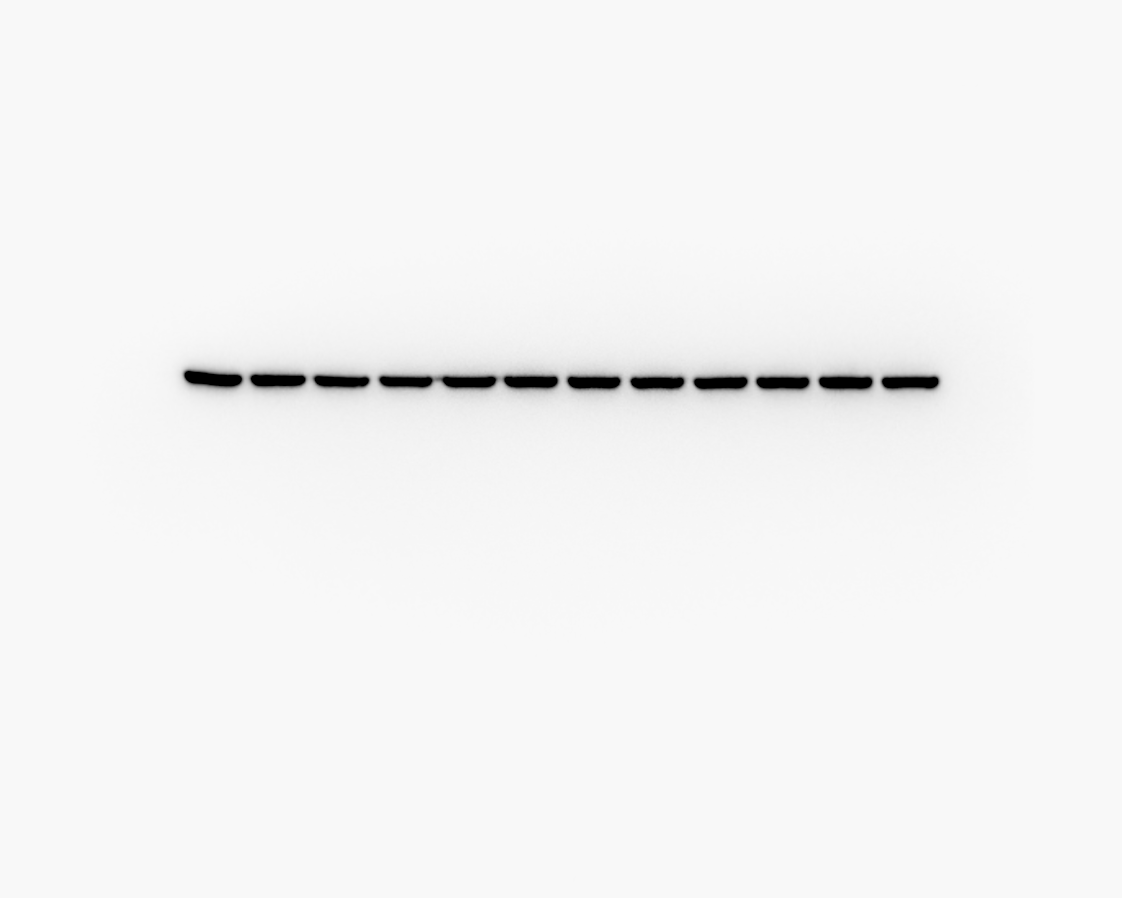

Supplement: Supplementary file 1 [file DataSheet1.zip › Image 1.TIF]

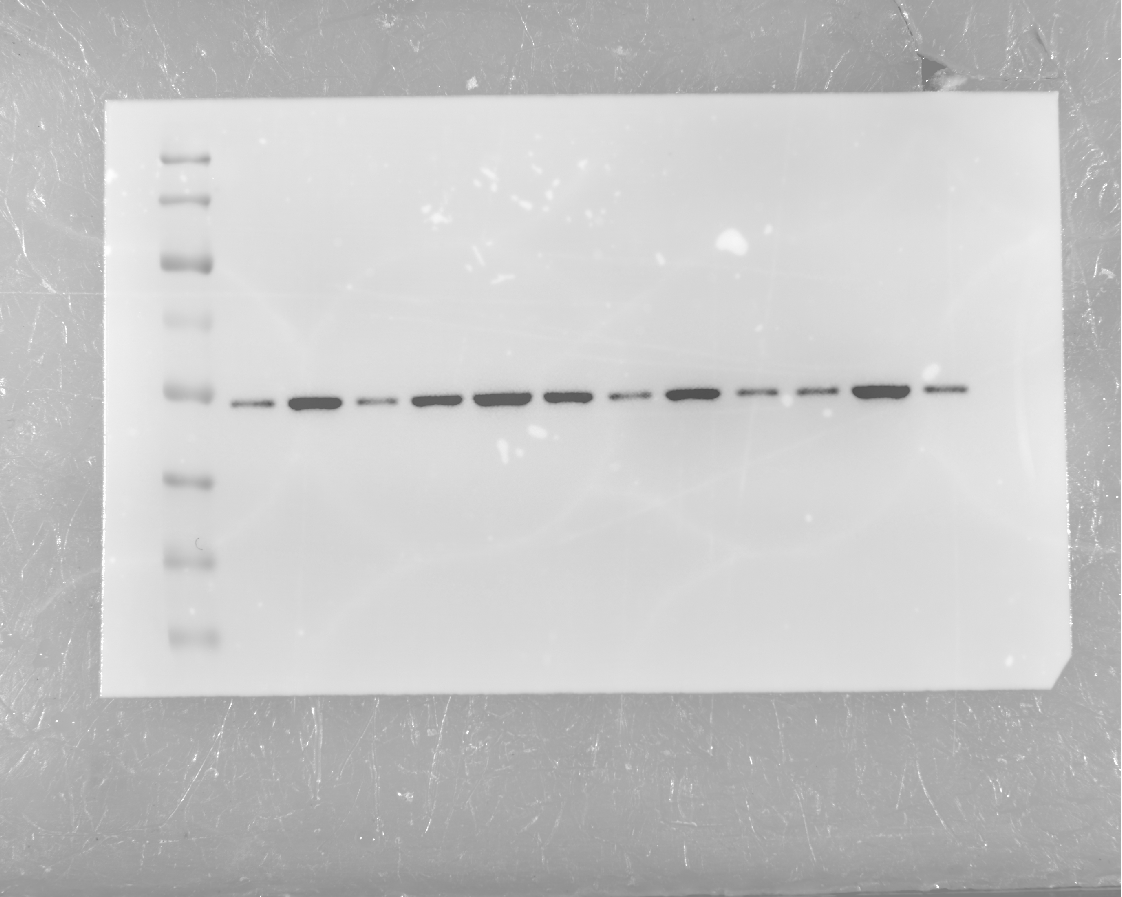

Supplement: Supplementary file 1 [file DataSheet1.zip › Image 10.TIF]

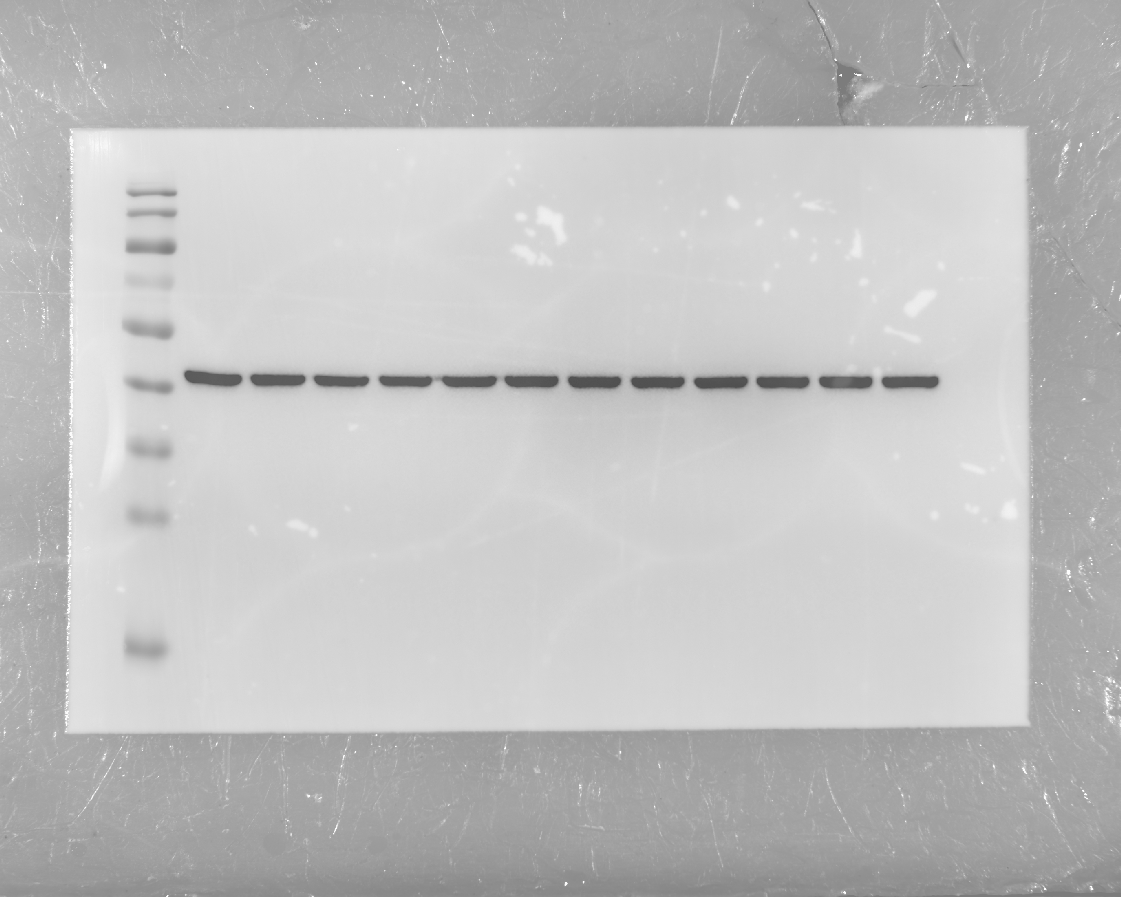

Supplement: Supplementary file 1 [file DataSheet1.zip › Image 2.TIF]

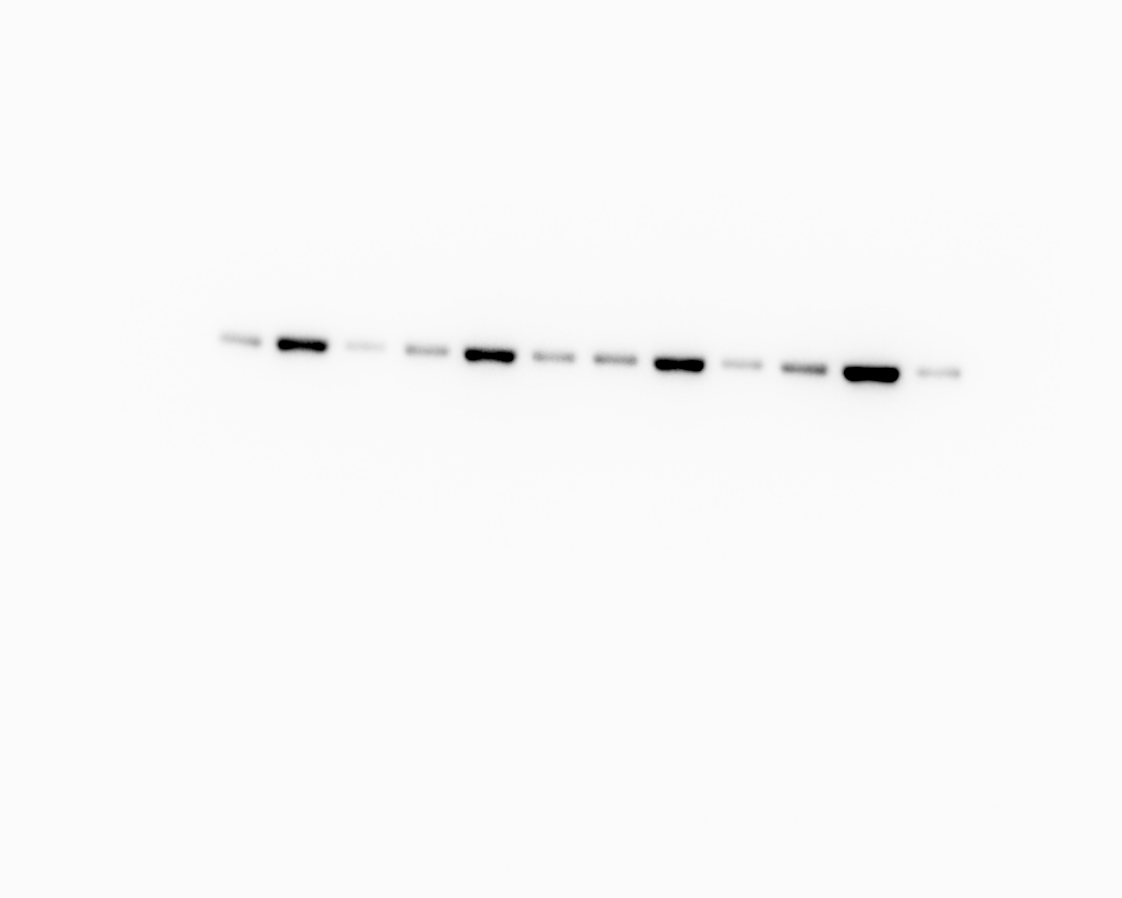

Supplement: Supplementary file 1 [file DataSheet1.zip › Image 3.TIF]

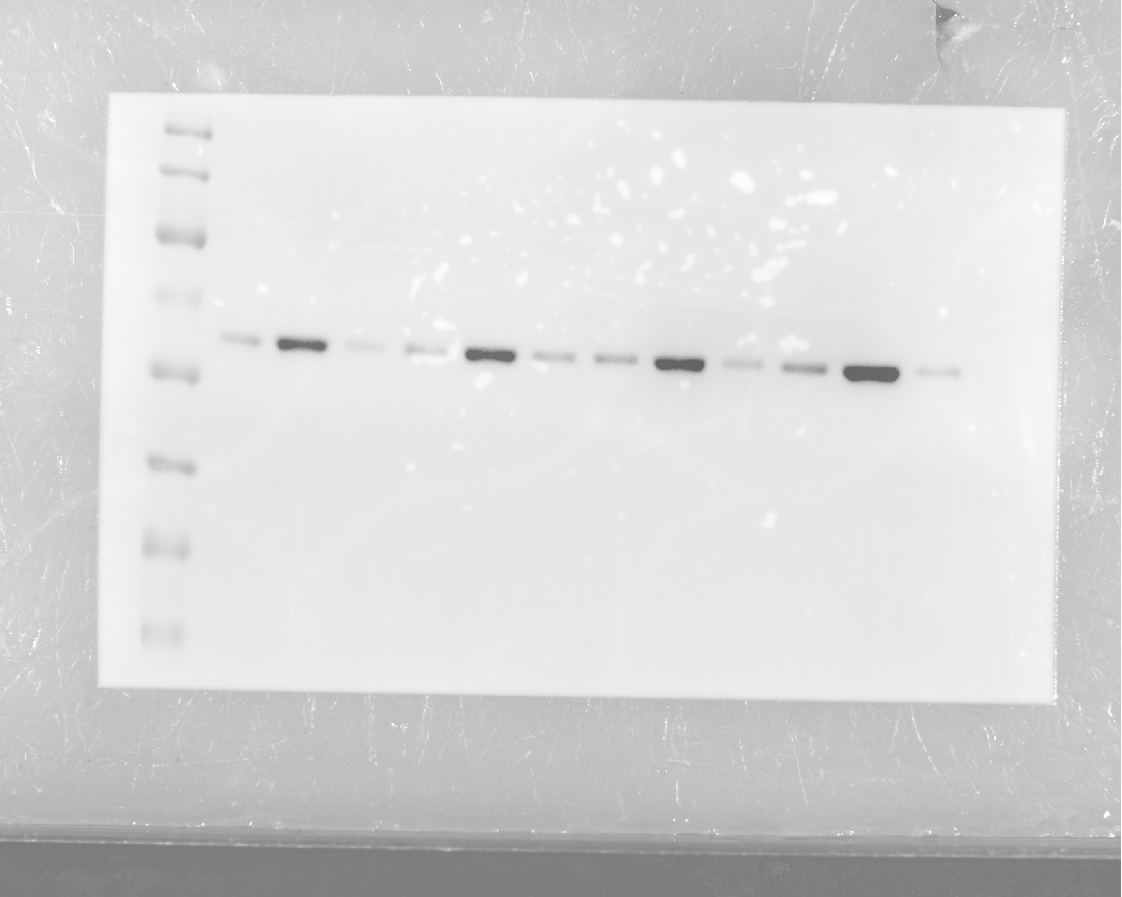

Supplement: Supplementary file 1 [file DataSheet1.zip › Image 4.TIF]

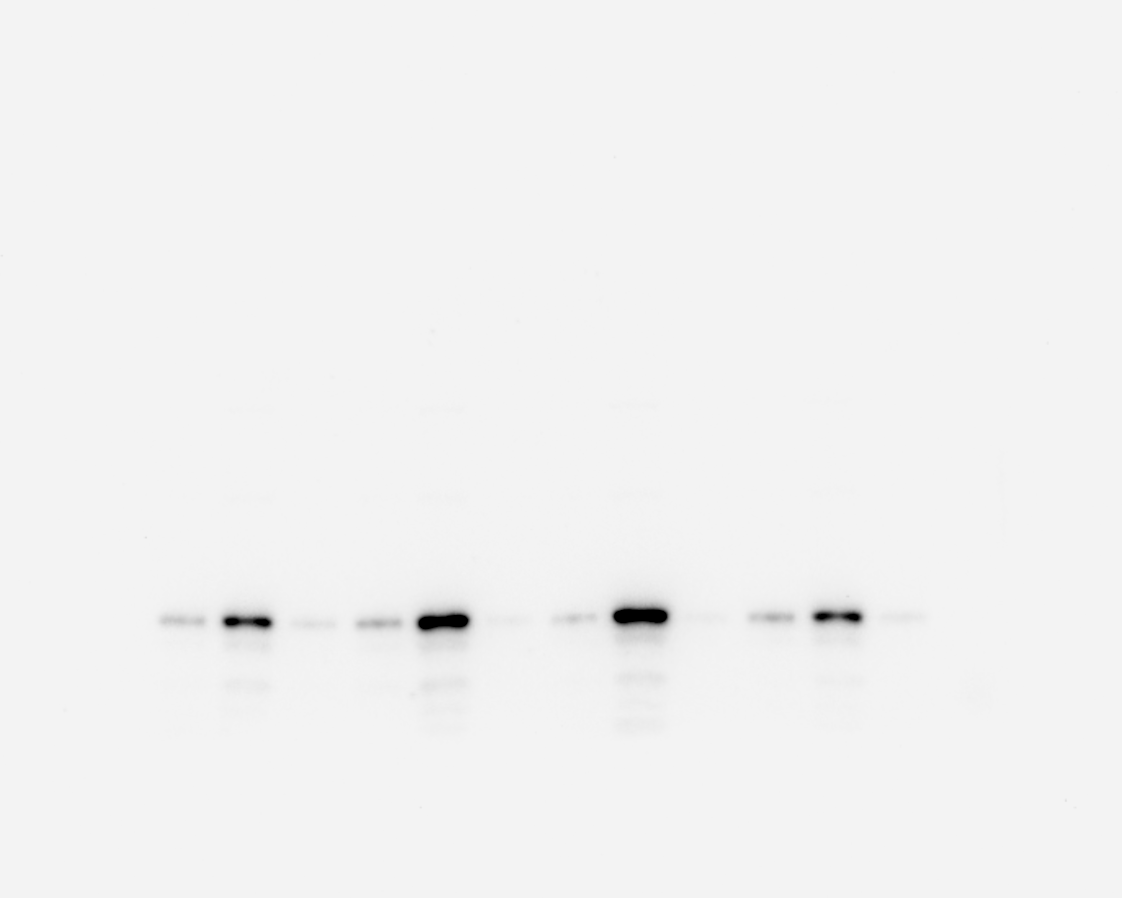

Supplement: Supplementary file 1 [file DataSheet1.zip › Image 5.TIF]

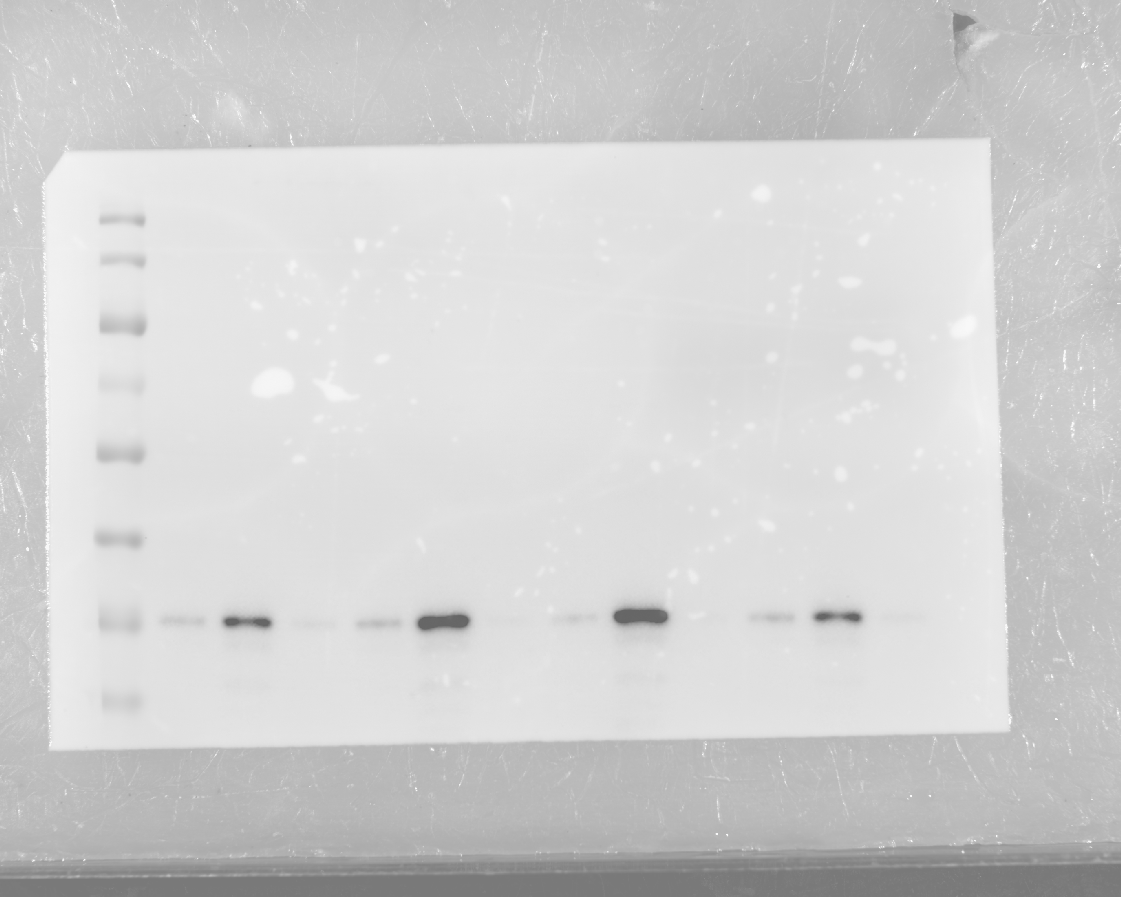

Supplement: Supplementary file 1 [file DataSheet1.zip › Image 6.TIF]

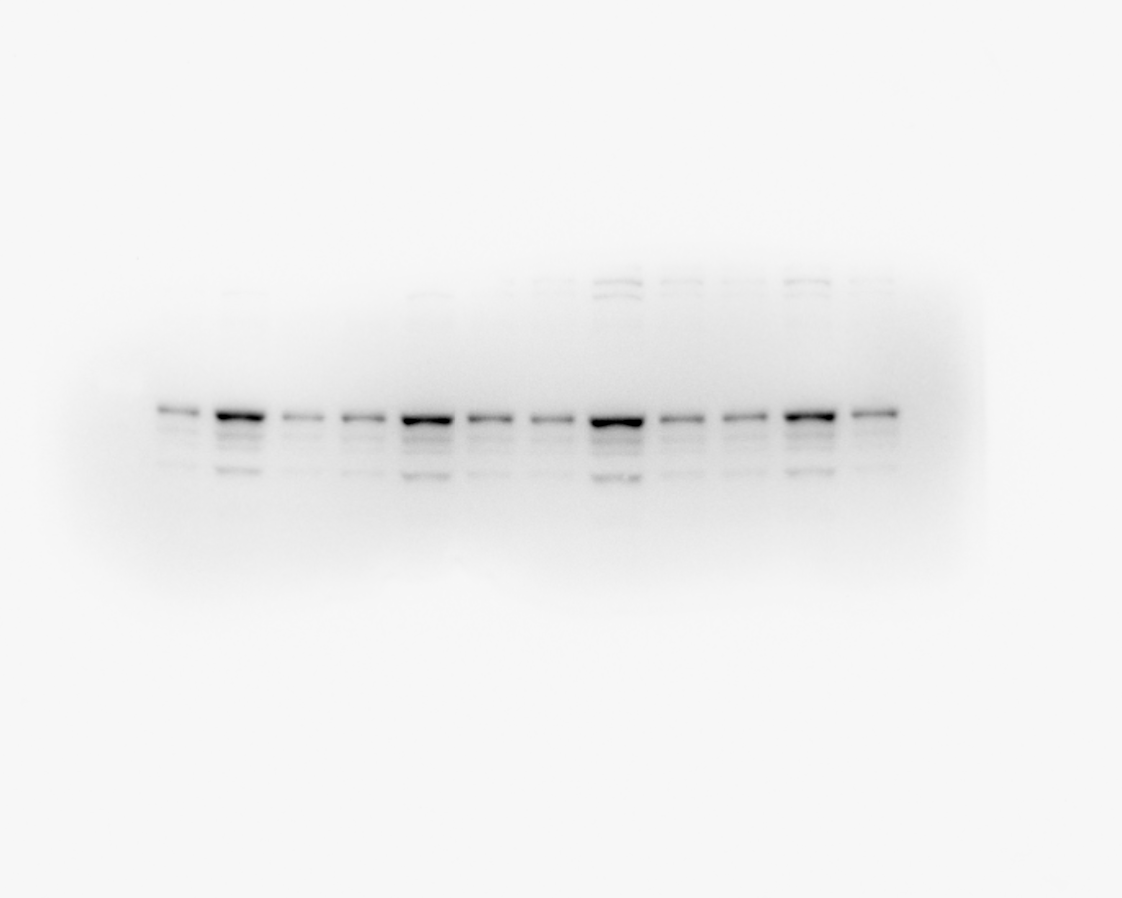

Supplement: Supplementary file 1 [file DataSheet1.zip › Image 7.TIF]

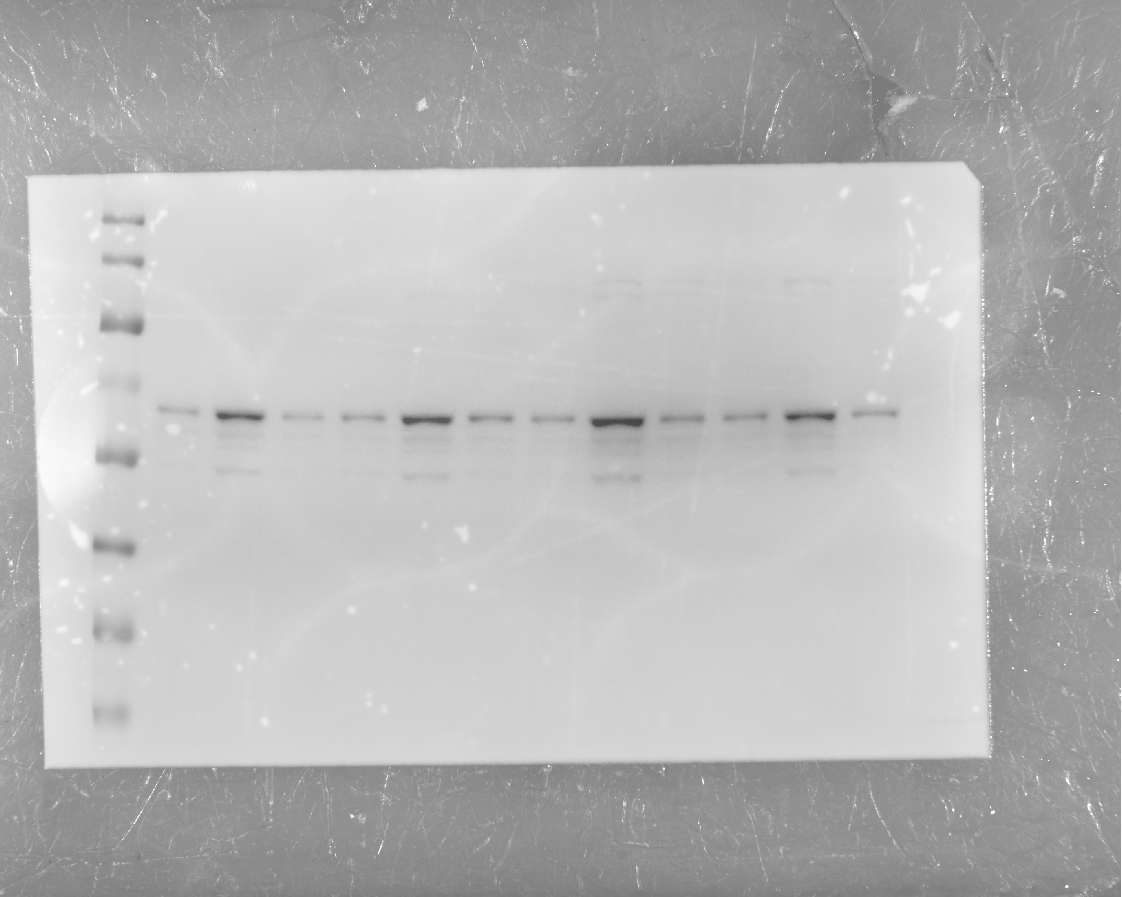

Supplement: Supplementary file 1 [file DataSheet1.zip › Image 8.TIF]

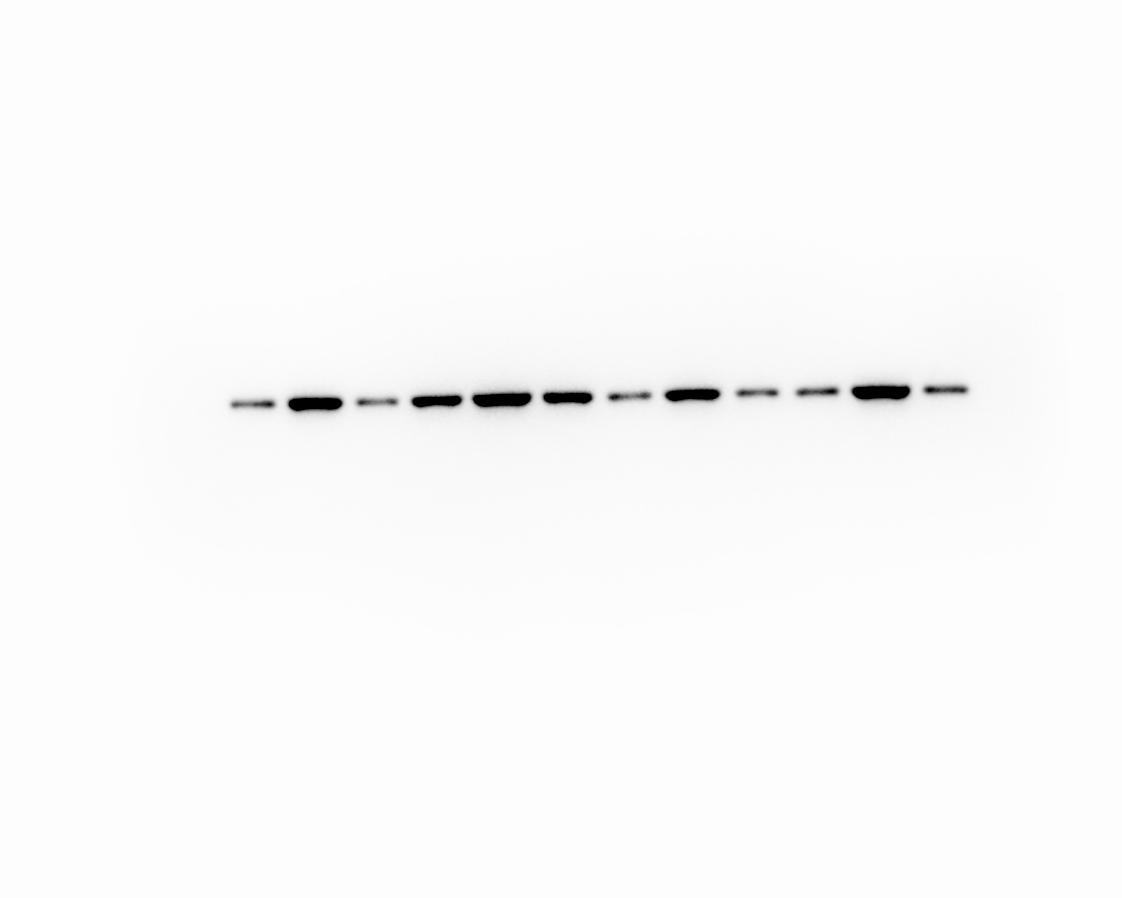

Supplement: Supplementary file 1 [file DataSheet1.zip › Image 9.TIF]
